# Percentile-Based Adaptive Immune Plasma Algorithm and Its Application to Engineering Optimization

**DOI:** 10.3390/biomimetics8060486

**Published:** 2023-10-14

**Authors:** Selcuk Aslan, Sercan Demirci, Tugrul Oktay, Erdal Yesilbas

**Affiliations:** 1 Department of Aeronautical Engineering, Erciyes University, Kayseri 38000, Turkey; 2 Department of Computer Engineering, Ondokuz Mayıs University, Samsun 55000, Turkey; 3 Air Safety Department, Qatar Civil Aviation Authority, Doha 122014, Qatar

**Keywords:** immune plasma algorithm, adaptive selection, percentile, big data, unmanned aerial vehicle, path planning

## Abstract

The immune plasma algorithm (IP algorithm or IPA) is one of the most recent meta-heuristic techniques and models the fundamental steps of immune or convalescent plasma treatment, attracting researchers’ attention once more with the COVID-19 pandemic. The IP algorithm determines the number of donors and the number of receivers when two specific control parameters are initialized and protects their values until the end of termination. However, determining which values are appropriate for the control parameters by adjusting the number of donors and receivers and guessing how they interact with each other are difficult tasks. In this study, we attempted to determine the number of plasma donors and receivers with an improved mechanism that depended on dividing the whole population into two sub-populations using a statistical measure known as the percentile and then a novel variant of the IPA called the percentile IPA (pIPA) was introduced. To investigate the performance of the pIPA, 22 numerical benchmark problems were solved by assigning different values to the control parameters of the algorithm. Moreover, two complex engineering problems, one of which required the filtering of noise from the recorded signal and the other the path planning of an unmanned aerial vehicle, were solved by the pIPA. Experimental studies showed that the percentile-based donor–receiver selection mechanism significantly contributed to the solving capabilities of the pIPA and helped it outperform well-known and state-of-art meta-heuristic algorithms.

## 1. Introduction

Easily implementable, relatively simple, flexible structures, gradient-free direction search mechanisms, and sufficient local minima-avoidance capabilities have increased the usage of meta-heuristics for solving different types of numerical or combinatorial optimization problems in recent years [1,2]. Even though there are various classification criteria for meta-heuristic algorithms, they are usually categorized by considering the kind of intelligence or phenomena to be modeled [3,4]. Meta-heuristics mimicking natural selection, crossover, mutation, or similar biological processes are generally referred to as evolutionary algorithms [5]. The genetic algorithm (GA) [6], differential evolution (DE) [7,8] algorithm, and evolutionary strategies (ES) [9] are the most famous evolutionary algorithms. Similar to these algorithms, population-based incremental learning (PBIL), proposed by Baluja, is another well-studied evolutionary technique that tries to empower the existing Darwinian operations with competitive learning [10]. The biogeography-based optimizer (BBO) proposed by Simon is also an evolutionary meta-heuristic that considers the distribution of biological species from one habitat to another via migration and how species arise and fade away to model the exploration and exploitation phases of a robust search [11,12].

The second group of meta-heuristics, also called swarm-intelligence (SI)-based meta-heuristics, considers the various behaviors of creatures such as ants, birds, bees, moths, bats, flowers, and even humans [13]. One of the most successful swarm-intelligence meta-heuristics is the ant colony (ACO) algorithm proposed by Dorigo and Caro [14]. The intelligent communication characteristics and food-source-finding capabilities of ants were used as a guide to design the ACO algorithm [14]. Particle swarm optimization (PSO) is another successful swarm-intelligence meta-heuristic in which the collective movements of bird blocking or fish schooling are referenced [15]. Krishnanand and Ghose tried to model how a glowworm attracts its companions, resulting in glowworm swarm optimization (GSO) [16]. The brood reproduction or parasitism of cuckoo birds gave inspiration to Yang and Deb, who introduced the cuckoo search (CS) algorithm [17]. The flashing nature of fireflies also gave inspiration to Yang in the development of the firefly algorithm (FA) [18]. The meta-heuristics introduced by Yang are not limited to the CS and FA. The bat algorithm (BA) [19], modeling the advanced echolocation properties of bats, and the flower pollination algorithm (FPA) [20], based on the self- and cross-pollination of flowers, was also announced in studies by Yang. The foraging habits of honeybees were analyzed by Karaboga, who presented the artificial bee colony algorithm (ABC for short) [21,22]. The gray wolf optimizer (GWO) algorithm was designed by Mirjalili et al. after investigating the hierarchy and hunting methods of gray wolves [23]. Mirjalili considered how moths navigate and fly at night and proposed the moth–flame optimization (MFO) algorithm [24]. Mirjalili also directly contributed to the development processes of the ant lion optimizer (ALO) [25], sine cosine algorithm (SCA) [26], multi-verse optimizer (MVO) [27], salp swarm algorithm (SSA) [28], Harris hawk optimizer (HHO) [29] and slime mold algorithm (SMA) [30]. Satapathy and Naik focused on the problem-solving concept of the social behavior of human beings, and social group optimization (SGO) was presented [31]. Tree social relations (TRS), introduced by Alimoradi et al. [32] after analyzing the collective and hierarchical life of trees, the gannet optimization algorithm (GOA) belonging to Pan et al. [33], developed based on the unique characteristics of foraging gannets, and the orchard algorithm (OA) developed by Kaveh et al. to model fruit-gardening procedures [34] are other recent meta-heuristics. The spotted hyena optimizer (SHO) [35], which mimics the collaborative hunting methods of the spotted hyena, the emperor penguin optimizer (EPO) [36], which was inspired by the huddling behavior of emperor penguins, and the seagull optimization algorithm (SOA) [37], which referenced how seagulls attack their prey, are recent competitive meta-heuristics proposed by Dhiman and Kumar. A special kind of sea bird called a sooty tern was investigated by Dhiman and Kaur, and the sooty tern optimization algorithm (STOA) was announced [38]. Although the migration behaviors of the abovementioned birds provided a steady exploration capability for the STOA, their spiral attacking method towards prey was modeled carefully to increase the exploitation capability of the same algorithm [38]. The tunicate swarm algorithm (TSA) is another SI-based meta-heuristic introduced as a result of studies by Dhiman and Kaur [39]. The main motivation behind the TSA was modeling the survival capacity of tunicates living in the depths of the ocean [39]. Experimental studies carried out with almost 100 test cases showed that the TSA is a strong optimizer and can be used successfully for different types of optimization problems [39].

Another group of meta-heuristics mainly focuses on using the fundamental steps of physical laws. Birbir and Fang proposed the electromagnetism-like algorithm (EMA), guided by the basics of electromagnetism [40]. The gravitational forces between masses became the source of motivation for Rashedi et al., and the gravitational search algorithm (GSA) was introduced [41]. Gravitational forces were interpreted by Formato differently, and the central force optimization (CFO) was developed [42]. The ray optimization (RO) algorithm, which simulates Snell’s law, describing the relationship between incident and reflected rays, was outlined by Shen and Li [43]. Cuevas et al. considered the transition between the solid, liquid, and gas phases of matter, and the state of matter search (SMS) was presented [44]. The interactions between positive and negative ions were referenced by Javidy et al. when the ions motion (IMO) algorithm was designed [45]. Savsani and Savsani focused on the mathematics of passing vehicles on a two-lane highway, and the passing vehicle search (PVS) was developed as a new meta-heuristic [46]. Azizi proposed the atomic orbital search (AOS) algorithm, for which the principles of quantum mechanics and quantum-based atomic schema related to the electron and nuclei were considered [47].

The intelligent behaviors of species, biological or evolutionary processes, and physical laws that have been tried to be modeled by the meta-heuristic algorithms are so diverse, as easily seen from the shortly summarized literature [48,49,50]. One of the most recent meta-heuristics showing how the natural phenomena guided by these problem-solving techniques can be various is the immune plasma algorithm (IP algorithm or IPA) [51]. IPA solves an optimization problem with its phases inspired by a medical method called immune or convalescent plasma treatment [51]. Even though the immune plasma treatment mainly depends on executing a relatively simple process, in which the antibody-rich part of the blood taken from the previously recovered patient or donor is transferred into the critical one or receiver, its efficiency and practical usage are proven again with the ongoing COVID-19 pandemic. In the standard implementation of the IPA, the number of donors and receivers are determined when control parameters are initialized, and remain unchanged until the end of the execution [51]. However, rather than assigning two different values to the number of donors and the number of receivers and guessing their interactions between them for each problem and running configuration, a simplified but effective method should be found and integrated into the workflow of the IPA. In this study, by considering this requirement about the IPA:

- A new donor and receiver selection mechanism based on a statistical metric known as the percentile was proposed.
- The new donor and receiver selection mechanism adjusted the number of donors and receivers in an adaptive manner due to the percentile description and the control parameters used in the standard IPA were not required.
- Because of the adaptive adjustment of donors and receivers at each infection cycle, the density of exploration and exploitation dominant operations were calibrated more robustly, and the solving capability increased.

The new IPA variant determining the number of donors and number of receivers with the proposed approach was called the percentile IPA, or pIPA. To analyze how the percentile-based selection mechanism affects the overall solving performance of the pIPA, a set of detailed experiments using 22 numerical benchmark problems and two challenging engineering problems, the former a big-data optimization problem requiring noise minimization and the latter a planning problem for an unmanned aerial system, was carried out. The detailed experiments and comparative studies showed that pIPA was capable of obtaining better solutions than other considered algorithms for most of the test cases. The rest of the paper is organized as follows: Fundamental properties of the IPA are summarized in Section 2. The newly proposed donor–receiver selection mechanism is introduced in Section 3. Details of the experimental studies, their results, and related interpretations are given in Section 4 and Section 5. Finally, the conclusion and possible works about the IPA and pIPA are presented in Section 6.

## 2. Immune Plasma Algorithm

The immune system is responsible for starting and managing a set of sophisticated defense operations with the lymphoid organs, T and B lymphocytes, to find and destroy antigens which are actually parasites, viruses, or part of them causing an infection [51]. The B lymphocytes or cells have receptors recognizing and binding specific antigens. When B lymphocytes bind to their specific antigens, they call upon T lymphocytes. T lymphocytes contribute to the multiplication of the B cells. In addition to this, T lymphocytes mature the B cells into plasma cells [51]. Plasma cells similar to T and B lymphocytes have an important role in the immune system. Each plasma cell is regulated for synthesizing an antigen-specific protein called an antibody [51]. Antibodies can be free-floating in the blood or seen on the membranes of different immune-system cells. Moreover, an antibody in both forms can bind its specific antigen to limit the interaction with this antigen and healthy cells [51]. Antibodies increase slowly with the start of an infection and reach a peak level [51]. However, in cases of immune-system diseases or disorders, the required number of antibodies cannot be produced. For infected individuals who are suffering from immune-system diseases or disorders, antibody-rich parts of the blood of the patients who have recovered shortly before can be a valuable source. Using the antibody-rich part of the blood, also called plasma, is the main motivation of the immune or convalescent plasma treatment [51] for critical patients. Even though the idea lying behind the immune plasma treatment is relatively simple, the efficiency of the biologically strong and evident implementation steps of the immune plasma treatment has been proven for the H1N1, SARS, MERS, Ebola, and the SARS-COV2, namely COVID-19 infection [51].

The properties of the immune plasma treatment were also guided by researchers, and a new meta-heuristic method known as the IP algorithm or IPA was proposed [51]. In the IP algorithm, each individual is assumed as a solution to the problem being solved. The immune-system response or level of antibody for an individual is directly matched with the quality or appropriateness of the solution in terms of objective function value [51]. The infection is distributed between individuals, and immune-system responses are determined. By controlling the immune-system responses of the individuals, while some of them are considered to be critical and become receivers, some of them become donors and contribute to the treatment operations of the critical individuals with their plasmas [51]. Until reaching a predetermined termination condition, the IP algorithm continues to spread infection between individuals for exploring the search space of the problem, select the donor and receiver individuals, and then apply plasma transfer to balance the exploration with the exploitation. The subsections given below describe the detailed workflow of the IP algorithm.

### 2.1. Details of Infection Distribution

The IP algorithm starts its operations by assigning initial values to the individuals in the population of size PS [51]. Assume that IPA tries to solve a *D*-dimensional problem. The initial value of the jth parameter related to the $x_{k}$ individual is calculated using Equation (1) [51]. In Equation (1), *k* is an index ranging from 1 to PS and the lower and upper bounds of the jth parameter are equal to the $x_{j}^{low}$ and $x_{j}^{high}$. Also, rand(0,1) corresponds to a random number generated between 0 and 1 for each calculation [51].

(1) $$x_{kj}=x_{j}^{low}+rand\left(0,1\right)\left(x_{j}^{high}-x_{j}^{low}\right)$$

An infection can easily distribute among individuals with droplets containing antigens. For describing how the randomly selected $x_{m}$ individual affects the $x_{k}$ and triggers the immune system, Equation (2) is employed by the IP algorithm [51]. Although the $x_{k}^{inf}$ represents the infectious $x_{k}$ individual, $x_{kj}^{inf}$ is used on behalf of the randomly selected jth parameter of $x_{k}^{inf}$ in Equation (2). It should be noted that the $x_{k}^{inf}$ individual is the same as the $x_{k}$ except the jth parameter. Also, $x_{kj}$ and $x_{mj}$ are matched with the jth parameters of the $x_{k}$ and $x_{m}$ individuals. Finally, rand(−1,1) is used on behalf of a random number between −1 and 1.

(2) $$\begin{array}{c} x_{kj}^{inf}=x_{kj}+rand\left(-1,1\right)\left(x_{kj}-x_{mj}\right)\\ where\\ m\in \{1,\ldots ,PS\}-\{k\} \end{array}$$

As stated earlier, immune-system responses or antibody levels of the individuals are directly matched with the corresponding objective function values. For a minimization problem with an objective function *f*, if the immune-system response or antibody level of the infectious $x_{k}$ individual showed as $f(x_{k}^{inf})$ is less than the immune-system response or antibody level of the same individual before the infection showed as $f(x_{k})$, it is assumed that $x_{k}$ recognizes the infection triggered by the $x_{m}$ and updates the immune system for the same or similar infection as in Equation (3) [51]. Otherwise, the immune system of the $x_{k}$ remains unchanged.

(3) $$x_{kj}=\left\{\begin{array}{cc} x_{kj}^{inf}, & if\;\;f\left(x_{k}^{inf}\right)<f\left(x_{k}\right)\\ x_{kj}, & otherwise \end{array}\right\}$$

### 2.2. Details of Plasma Treatment

After infecting all individuals in the population, IPA first decides how many individuals will be donors and how many individuals will be receivers. For this purpose, it describes two different control parameters known as NoR and NoD [51]. Although NoR is matched with the abbreviation of the number of receivers, NoD represents the abbreviation of the number of donors. The values of the NoR and NoD parameters are assigned when the IPA is initialized, and the first NoR worst individuals are treated with the plasmas of the first NoD best individuals [51]. If $x_{k}^{rcv}$ is the *k* indexed receiver in the set of receivers of size NoR and $x_{m}^{dnr}$ is the randomly determined donor in the set of donors of size NoD, the plasma of $x_{m}^{dnr}$ is transferred into the $x_{k}^{rcv}$ using Equation (4) given below [51]. In Equation (4), $x_{kj}^{rcv}$ and $x_{mj}^{dnr}$ are matched with the jth parameters of the $x_{k}^{rcv}$ and $x_{m}^{dnr}$ individuals. Furthermore, $x_{k}^{rcv-p}$ represents the plasma transferred $x_{k}^{rcv}$ and its jth parameter is the $x_{kj}^{rcv-p}$. It should be noted that each parameter of the $x_{k}^{rcv}$ is modified with the corresponding parameter of the $x_{m}^{dnr}$ by guiding Equation (4).

(4) $$\begin{array}{c} x_{kj}^{rcv-p}=x_{kj}^{rcv}+rand\left(-1,1\right)\left(x_{kj}^{rcv}-x_{mj}^{dnr}\right)\\ where\\ j\in \{1,\ldots ,D\} \end{array}$$

The immune-system response or antibody level of the $x_{k}^{rcv}$ after the first dose of plasma is important for deciding whether the second dose of the plasma will be transferred or not. If $f(x_{k}^{rcv-p})$ showing the antibody level of $x_{k}^{rcv}$ after the first dose plasma is less than the $f(x_{m}^{dnr})$, $x_{k}^{rcv}$ is changed with $x_{k}^{rcv-p}$ and second dose of plasma from $x_{m}^{dnr}$ is prepared for transferring. Otherwise, $x_{k}^{rcv}$ is changed with the $x_{m}^{dnr}$ to guarantee that a single plasma dose is given and the treatment is ended [51]. When the IP algorithm decides to apply the second dose of plasma, it first determines the new $x_{k}^{rcv-p}$ and then compares $f(x_{k}^{rcv-p})$ showing the antibody level of $x_{k}^{rcv}$ after the second dose plasma with the $f(x_{k}^{rcv})$ value of the current $x_{k}^{rcv}$. If $f(x_{k}^{rcv-p})$ is less than $f(x_{k}^{rcv})$, $x_{k}^{rcv}$ is changed with $x_{k}^{rcv-p}$ and third dose of plasma from $x_{m}^{dnr}$ is prepared for transferring. Otherwise, the plasma treatment is ended for the $x_{k}^{rcv}$ [51].

The IP algorithm controls and modifies the immune memories of the donors by considering the ratio between $t_{cr}$ and $t_{max}$ after the plasma treatment is completed for all receivers. Although $t_{cr}$ shows the current evaluation or calculation number of the objective function, $t_{max}$ corresponds to the maximum evaluation or calculation number of the objective function. If a random number produced between 0 and 1 is less than $t_{cr}/t_{max}$, each parameter of the $x_{m}^{dnr}$ where *m* ranges from 1 to NoD is updated using Equation (5) [51]. If the mentioned random number is equal or higher than $t_{cr}/t_{max}$, each parameter of the $x_{m}^{dnr}$ is initialized with Equation (1) [51]. As easily seen from the model used to control and update the donors, it is understood that the probability of producing a random number less than $t_{cr}/t_{max}$ increases, and the $x_{m}^{dnr}$ donor is modified slightly as in Equation (5) [51].

(5) $$x_{mj}^{dnr}=x_{mj}^{dnr}+rand\left(-1,1\right)x_{mj}^{dnr}$$

## 3. Modified Donor–Receiver Selection Mechanism

The standard implementation of IPA determines the number of donors and number of receivers at initialization, and their values are protected until the end of execution. Selecting the first NoD best individual or individuals from the population and using them as plasma sources for the first NoR worst individual or individuals significantly contributed to the local search capability of the IPA, and experimental studies showed that the value of the NoD parameter should be chosen equal or less than the value of the NoR parameter [51]. However, it should be noted that determining the appropriate values for both NoD and NoR parameters and guessing their effect on the solving capabilities of the algorithm and interactions between them are difficult. Rather than assigning static values to the NoD and NoR parameters, a more convenient, implicitly self-adjustable, and simplified approach by considering the existing control parameters such as NoD and NoR can be proposed and integrated into the workflow of the IP algorithm.

Percentile is one of the most commonly used metrics in order statistics [52]. It helps to indicate where a special value falls within a distribution of a set of values and understand the relative standing of that value. Assume that the *x* element is at the $k^{th}$ percentile. By considering this assumption, it is said that the *x* element is greater than the k% of the other elements related to the same set. In addition to the help of evaluation the relative standing of a given element within a distribution, percentile also provides a method for dividing the dataset into partitions [53]. If the *x* element is at the $k^{th}$ percentile, the dataset is divided into two partitions. Although the first partition contains the *k* percent of the whole dataset whose elements are less than *x*, the second partition contains the rest of the initial dataset, and each element in the second partition is equal to or greater than *x*. For deciding which element in this dataset will be chosen as *x* and will be at the $k^{th}$ percentile, elements of the dataset are first sorted in ascending order, and then Equation (6) given below is utilized [54]. In Equation (6), *N* shows the number of elements in the dataset, and *r* corresponds to the index or rank of the element that will be chosen from the sorted dataset on behalf of *x* and at the $k^{th}$ percentile.

(6) $$r=\lceil \frac{k}{100}\times N\rceil$$

**Figure 1:**
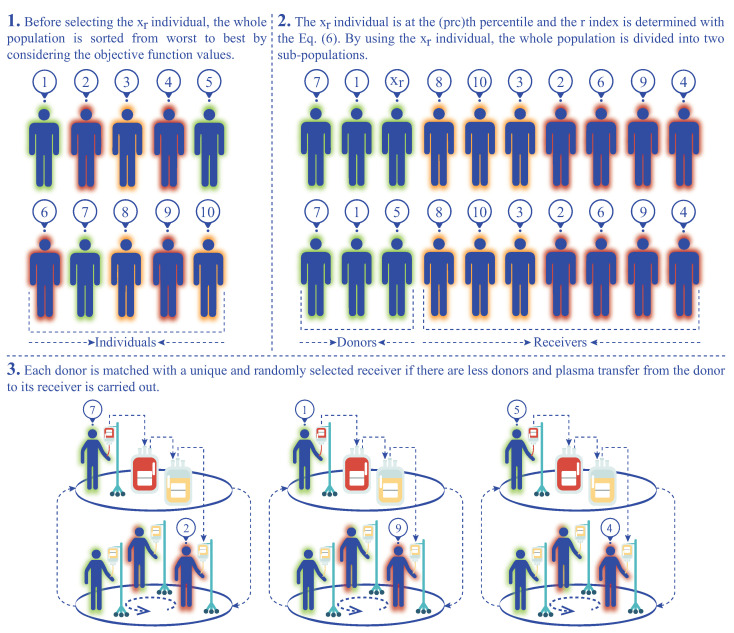
Workflow of the percentile-based donor–receiver selection strategy.

When considering the properties of the percentile metric about the intrinsic division of the dataset, it is seen that the population of the IP algorithm can be partitioned into two groups using the percentile calculation. One of the groups is devoted to the possible donor or donors, while the other is related to the possible receiver or receivers, and then a new variant of the IP algorithm named percentile IP algorithm (pIPA) can be introduced. In the pIPA, rather than determining the number of donors and the number of receivers separately by assigning values to both NoD and NoR control parameters, the possible donor and receiver individuals are determined with a new and single control parameter showed as prc. The prc parameter is actually used to find the individual that is at the $prc^{th}$ percentile. For a minimization problem, pIPA first sorts the individuals in the population of size PS according to their objective function values, and the *r* index and $x_{r}$ individual are determined as in Equation (6) by changing the *k* with the prc. If the objective function value of the $x_{i}$ individual where *i* ranges from 1 to PS is less than or equal to the objective function value of the $x_{r}$, the $x_{i}$ individual becomes a donor candidate and it is added into the set of donors. Otherwise, $x_{i}$ individual becomes a receiver candidate, and it is added to the set of receivers. By considering the relationship between the $x_{r}$ and other individuals in the population, it is seen that nearly prc percent of the population becomes the receiver candidates, and (100−prc) percent of the population becomes the donor candidates. The workflow of the percentile-based donor–receiver selection strategy can be summarized visually as in Figure 1.

After generating the set of donors and set of receivers by utilizing the value assigned to the prc parameter, pIPA controls the number of possible donors and receivers. If there are more donors compared to the receivers, each receiver is matched with a unique and randomly selected donor, and plasma transfer is carried out for the receivers as per the standard IPA. In Algorithm 1, the plasma transfer operations are summarized by considering that there are more donors than the receivers. If there are more receivers compared to the donors, each donor is matched with a unique and randomly selected receiver, and plasma transfer from the donor to its receiver is carried out as in the standard IP algorithm. Algorithm 2 states the donor and receiver selection and plasma transfer operations of the pIPA for the scenarios in which there are more receivers than the donors or there is an equal number of donors and receivers.

Even though the value being assigned to the prc remains unchanged until the end of the execution, the number of possible donors and receivers can vary because of the description of the percentile and objective function values of individuals in the population. If there are individuals whose objective function values are the same as the objective function value of the $x_{r}$, more than (100−prc) percent of the population can be related to the set of possible donors. As an expected result of this situation, less then prc percent of the population can be related to the set of possible receivers, and the number of donors and number of receivers can be adjusted dynamically. Another important situation that should be considered is the equivalence of the $x_{r}$ individual with the remaining individuals of the population based on the objective function values. If the objective function value of $x_{r}$ is equal to the objective function values of the remaining individuals in the population, the set of possible receivers is empty and pIPA continues spreading infection without applying plasma transfer operations.
**Algorithm 1** Plasma transfer in the pIPA by selecting donors1:$x_{prc}\leftarrow$ find required individual2:dCount← get the number of donors3:rCount← get the number of receivers4:$x_{best}\leftarrow$ get the best individual found so far5:**if** dCount>rCount **then**6:    doseControl[1…rCount]← set elements to 17:    treatmentControl[1…rCount]← set elements to 18:    rIndexes[1…rCount]← get the indexes of the receivers9:    dIndexes[1…rCount]← get the indexes of chosen donors10:    **for** i←1⋯rCount **do**11:        $x_{m}^{dnr}\leftarrow$ get the dIndexes[i] indexed donor12:        $x_{k}^{rcv}\leftarrow$ get the rIndexes[i] indexed receiver13:        **while** treatmentControl[i]==1 **do**14:           **if** $t_{cr}<t_{max}$ **then**15:               $t_{cr}\leftarrow t_{cr}+1$16:               $x_{k}^{rcv-p}\leftarrow$ treatment of $x_{k}^{rcv}$ with $x_{m}^{dnr}$17:               **if** doseControl[i]==1 **then**18:                   **if** $f\left(x_{k}^{rcv-p}\right)<f\left(x_{m}^{dnr}\right)$ **then**19:                       Update $x_{k}^{rcv}$ with $x_{k}^{rcv-p}$20:                       doseControl[i]←doseControl[i]+121:                   **else**22:                       Update $x_{k}^{rcv}$ with $x_{m}^{dnr}$23:                       treatmentControl[i]←024:                   **end if**25:               **else**26:                   **if** $f\left(x_{k}^{rcv-p}\right)<f\left(x_{k}^{rcv}\right)$ **then**27:                       Update $x_{k}^{rcv}$ with $x_{k}^{rcv-p}$28:                   **else**29:                       treatmentControl[i]←030:                   **end if**31:               **end if**32:               **if** $f\left(x_{k}^{rcv}\right)<f\left(x_{best}\right)$ **then**33:                   Update $x_{best}$ with $x_{k}^{rcv}$34:               **end if**35:           **end if**36:        **end while**37:    **end for**38:**end if**

**Algorithm 2** Plasma transfer in the pIPA by selecting receivers
1:$x_{prc}\leftarrow$ find required individual2:dCount← get the number of donors3:rCount← get the number of receivers4:$x_{best}\leftarrow$ get the best individual found so far5:**if** dCount<=rCount **then**6:    doseControl[1…dCount]← set elements to 17:    treatmentControl[1…dCount]← set elements to 18:    dIndexes[1…dCount]← get the indexes of the donors9:    rIndexes[1…dCount]← get the indexes of chosen receivers10:    **for** i←1⋯dCount **do**11:        $x_{m}^{dnr}\leftarrow$ get the dIndexes[i] indexed donor12:        $x_{k}^{rcv}\leftarrow$ get the rIndexes[i] indexed receiver13:        **while** treatmentControl[i]==1 **do**14:           **if** $t_{cr}<t_{max}$ **then**15:               $t_{cr}\leftarrow t_{cr}+1$16:               $x_{k}^{rcv-p}\leftarrow$ treatment of $x_{k}^{rcv}$ with $x_{m}^{dnr}$17:               **if** doseControl[i]==1 **then**18:                   **if** $f\left(x_{k}^{rcv-p}\right)<f\left(x_{m}^{dnr}\right)$ **then**19:                       Update $x_{k}^{rcv}$ with $x_{k}^{rcv-p}$20:                       doseControl[i]←doseControl[i]+121:                   **else**22:                       Update $x_{k}^{rcv}$ with $x_{m}^{dnr}$23:                       treatmentControl[i]←024:                   **end if**25:               **else**26:                   **if** $f\left(x_{k}^{rcv-p}\right)<f\left(x_{k}^{rcv}\right)$ **then**27:                       Update $x_{k}^{rcv}$ with $x_{k}^{rcv-p}$28:                   **else**29:                       treatmentControl[i]←030:                   **end if**31:               **end if**32:               **if** $f\left(x_{k}^{rcv}\right)<f\left(x_{best}\right)$ **then**33:                   Update $x_{best}$ with $x_{k}^{rcv}$34:               **end if**35:           **end if**36:        **end while**37:    **end for**38:
**end if**



Similar to the standard implementation of IPA, pIPA also differs from most meta-heuristics when the number of evaluations or the number of objective function calls per cycle, iteration, or generation is considered. The NoD and NoR parameters of the standard IPA and prc parameter of the pIPA can change the number of evaluations from one cycle to another because of the possible repetition of the plasma treatment for receivers. Even though the number of evaluations consumed per cycle changes in both IPA and pIPA, they complete their operations if the maximum evaluation number abbreviated as $t_{max}$ in the previous section is reached. When the IPA and pIPA are employed in order to solve a *D*-dimensional problem for which the complexity of calculating the objective function is estimated as O(D) using big-O notation, the running time of the IPA and pIPA becomes equal to $O(t_{max}\times D)$. This description of the running time of the IPA and pIPA in terms of $t_{max}$ and the complexity of the objective function calculation can be guided for comparison with other meta-heuristics.

A more specialized analysis for the running time of the pIPA can be made by considering the cost of newly added or existing operations such as distribution of infection, selection of the $x_{r}$ individual, treatment of the receiver or receivers and modification of the donor or donors. When pIPA with PS individuals starts solving a *D*-dimensional problem for which the complexity of calculating the objective function is O(D), the cost stemmed from the distribution of infection is found as O(PS×D). After completing the distribution of infection, pIPA divides the whole population by considering the value assigned to the prc. All individuals are first sorted by a sorting algorithm for which the complexity is equal to $O(PS\times log_{2}PS)$ and the number of possible receivers shown as Rc, and the number of possible donors shown as Dn are determined. If the Rc is equal to zero, i.e., there is no receiver in the population, the complexity of this cycle is defined as $O(PS\times \left(D+log_{2}PS\right))$. Otherwise, the cost of giving one dose plasma for each receiver and modification of the donor or donors are found as $O(Rc\times D^{2}+Dn\times D^{2})$ and the running time of the pIPA for a cycle becomes equal to $O(PS\times D+PS\times logPS+D^{2}\times \left(Rc+Dn\right))$. Because the sum of Rc and Dn is equal to PS, the running time of the pIPA can be shown as $O(PS\times \left(D+logPS+D^{2}\right))$ or simply $O(PS\times D^{2})$ by utilizing from the property of the used asymptotic notation and the dominance of $D^{2}$ term.

## 4. Experimental Studies

The possible contribution of the percentile-based donor–receiver selection strategy can vary with the values assigned to the control parameters such as population size, maximum evaluation number, dimensions, prc, and types of optimization problems. To provide a clear vision of how the newly proposed strategy changes the solving capabilities, experimental studies were divided into four major subsections. In the first subsection, 100 and 200-dimensional classical numerical problems were solved with the pIPA by assigning different values to the prc. Obtained results by the pIPA were also compared with a set of meta-heuristics including IPA [51], PSO [55], GSA [41], CS [17], BA [19], FPA [20], SMS [44], FA [18], GA [6], MFO [24] and ALO [25]. The second subsection of the experimental studies was devoted to the investigation of the capabilities of pIPA in solving complex numerical problems first introduced at the CEC 2015. The results of the pIPA for CEC 2015 benchmark problems were compared with the IPA [51], SOA [37], SHO [35], GWO [23], PSO [55], MFO [24], MVO [27], SCA [26], GSA [41], GA [6] and DE [56]. In the third subsection of the experimental studies, a recent real-world engineering problem that requires splitting a source signal into noise and noise-free parts optimally was solved with pIPA, and comparisons between pIPA and other well-known meta-heuristics such as IPA [51], GA [6], PSO [55], DE [56], ABC [57], GSA [41], MFO [24], SCA [26], SSA [28] and HHO [29] were carried out. In the last subsection, pIPA was also used to solve another real-world problem for which the path of an unmanned aerial vehicle (UAV) or an unmanned combat aerial vehicle (UCAV) is tried to be determined by considering the enemy threats and fuel consumption. The results of the pIPA for path planning problem were compared with the IPA [51], BA [58], BAM [58], ACO [59], BBO [59], DE [59], ES [59], FA [59,60], GA [59], MFA [59,60], PBIL [59], PSO [59], SGA [59] and PGSO [59]-based approaches.

### 4.1. Solving Classical Benchmark Problems with pIPA

The benchmark problems or functions for which the formulation, lower and upper bounds are given in Table 1 were solved with the pIPA. The global minimum values of all these problems except the $f_{8}$ are equal to zero. For the $f_{8}$, the global minimum value is calculated as −D×418.98 where *D* corresponds to the number parameters as stated before. When solving the 100-dimensional benchmark problems given in Table 1, the population size and maximum evaluation number were set to 30 and 30,000 [24]. To analyze how the qualities of the solutions change with the values assigned to prc, nine positive integers, including 30, 35, 40, 50, 60, 70, 80, 90, and 95 were used. The pIPA with the mentioned configurations was tested 30 times for each problem instance using random seeds. The objective function values of the best solutions found at each of 30 runs were averaged and reported in Table 2 with the related standard deviations.

The results reported in Table 2 give important information about the pIPA and appropriate values of the prc parameter. Although the pIPA obtains the global minimum solutions with all of the nine different values assigned to the prc for the $f_{6}$, $f_{9}$ and $f_{11}$ functions, it finds relatively close mean best objective function values for the $f_{5}$ function with all of the constants assigned to the prc. As distinct from the global minimums of $f_{6}$, $f_{9}$ and $f_{11}$ functions, the global minimum of the $f_{5}$ function is located at the end of a long and narrow valley and converging to the global minimum of it is extremely difficult. Because of this main reason, meta-heuristic algorithms usually require subtly configured control parameters and more evaluations for the mentioned function. However, it should be noted that the percentile-based donor–receiver selection mechanism adjusts the workflow and contributes to the convergence of the pIPA even though the prc is changed. For $f_{1}$, $f_{2}$, $f_{8}$ and $f_{12}$ functions, the qualities of the solutions found by the pIPA get better or change slightly when the value assigned to the prc increases from 30 to 80 or even 90. Similar generalizations can also be made for the $f_{3}$, $f_{4}$, $f_{7}$, and $f_{10}$ functions by considering a small set of values of prc. The qualities of the solutions found by the pIPA get better or change slightly for the these functions when the prc increases from 30 to 40.

As stated before, the number of donors and receivers in pIPA can be different at each cycle, while the prc remains unchanged. To analyze whether the number of donors and the number of receivers change or not when the initial value of the prc is preserved until the end of a run, they are first counted at each cycle, averaged, and then recorded. After completing 30 independent runs, the number of donors and number of receivers recorded for each run are averaged again and presented in Table 3. When the results given in Table 3 are controlled, it is seen that the newly proposed mechanism is capable of changing or adjusting the number of donors and receivers for the $f_{4}$, $f_{6}$, $f_{9}$, $f_{10}$ and $f_{11}$ functions. It tries to increase the number of donors and decrease the number of receivers while the value of the prc is protected. By changing the number of donors and number of receivers without increasing or decreasing the initial value of the prc, pIPA also has a chance to adjust the execution of exploration and exploitation dominant phases explicitly.

The newly proposed percentile-based donor–receiver selection strategy requires the execution of extra computational operations compared to the standard implementation of the IPA and changes the density of the exploration and exploitation dominant phases. To understand whether the usage of the percentile-based donor–receiver selection strategy increases the execution time of the algorithm or not, the duration of each run in terms of seconds is recorded and then averaged after the completion of 30 independent tests of pIPA with different prc values. Also, the duration of each run in terms of seconds is recorded and then averaged when 30 independent tests are completed for the standard IPA whose NoD and NoR parameters are set to 1. Both pIPA and IPA were coded in C programming language, and experiments were carried out on a PC equipped with a single-core processor with 1.33 Ghz.

From the average execution times and related standard deviations belonging to pIPA and IPA given in Table 4, it is clearly seen that IPA requires less time compared to the pIPA with lower prc values. Moreover, it is understood that there is a relationship between the execution time of the pIPA and the value assigned to the prc. Although the prc is increased, the average execution time of the pIPA generally decreases. If the prc is increased, the number of possible donors decreases, and plasma treatment is carried out for a small set of randomly determined receivers. Otherwise, the number of possible donors increases, more receivers are supported with the plasma treatment, and the execution time of the pIPA increases because of the computationally intensive operations of the plasma transfer. However, it should be noted that when the difference between the number of donors tried to be adjusted with the prc and NoD decreases, the difference between the average execution times of the pIPA and IPA also decreases.

The contribution of the proposed mechanism can be understood by comparing the results of the pIPA with the results of other meta-heuristics. For this purpose, the results of the pIPA were compared with the results of the IPA [51], MFO [24], PSO [55], GSA [41], BA [19], FPA [20], SMS [44], FA [18] and GA [6]. To guarantee that all meta-heuristics obtain their results under the same conditions, population sizes of them were set to 30, and the maximum evaluation number was taken equal to 30,000 [24,51]. Although the prc of the pIPA was 90 for $f_{1}$, $f_{2}$, $f_{6}$, $f_{8}$, $f_{9}$ and $f_{11}$, it was determined as 60 and 50 for the $f_{5}$ and $f_{12}$. Moreover, the value of the prc was equal to 40 for $f_{3}$, $f_{7}$ and $f_{10}$ and equal to 30 for the $f_{4}$ function. When the mean best objective function values and standard deviations belonging to the 30 independent runs of these algorithms in Table 5 are investigated, the superiority of the pIPA can be seen. For 10 of 12 benchmark functions, pIPA outperforms its competitors or obtains the same mean best objective function values. It only lags behind the IPA for the $f_{8}$ function and the GSA for the $f_{12}$ function and becomes the second-best algorithm among other tested meta-heuristics for these functions. The idea lying behind the pIPA manages donor and receiver selection operations more robustly compared to the standard implementation of the IPA by setting only one control parameter. In pIPA, the number of donors and receivers can be updated from one cycle to another while the prc remains unchanged. Furthermore, although the number of donors and receivers are the same for different cycles, donors and receivers are matched by a controlled–randomized approach, and receivers have a chance of treatment with the plasma of a different donor.

**Figure 2:**
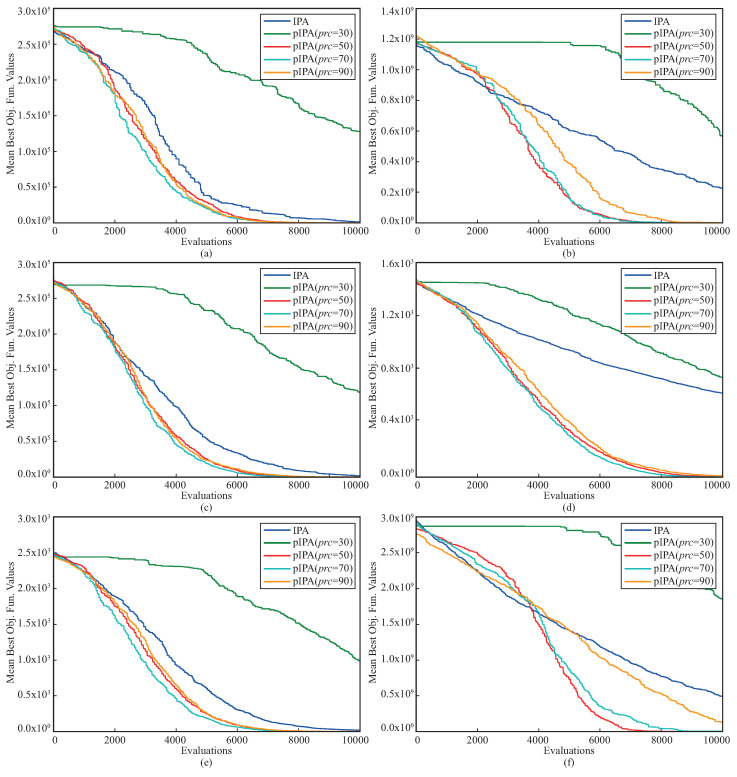
Convergence curves of pIPA and IPA for $f_{1}$ (**a**), $f_{5}$ (**b**), $f_{6}$ (**c**), $f_{9}$ (**d**), $f_{11}$ (**e**) and $f_{12}$ (**f**) functions.

Another comparison between pIPA and IPA was made for the convergence performances. To analyze and compare the convergence performances of meta-heuristic algorithms, there are two commonly used metrics, namely success rate and mean evaluation. If a run of the algorithm achieves a better solution compared to a threshold before the previously determined termination criteria are met, it is said that the run is successful. The success rate is the ratio between the number of successful runs and the total number of runs. For each successful run, the minimum number of function evaluations required to achieve a better solution compared to a threshold is recorded. The average of these recorded values corresponds to the mean evaluation. The convergence comparison between pIPA and IPA was made by setting the threshold to $1\times 10^{-25}$ for $f_{1}$, $f_{2}$, $f_{3}$, $f_{6}$, $f_{9}$ and $f_{11}$ functions, $1\times 10^{-10}$ for $f_{10}$ function, $1\times 10^{-03}$ for $f_{7}$ function, $1\times 10^{00}$ for $f_{12}$ function, $1\times 10^{01}$ for $f_{4}$ function, $1\times 10^{02}$ for $f_{5}$ function, $-1\times 10^{04}$ for $f_{8}$ function and then success rate and mean evaluation metrics abbreviated as Sr and Me were summarized in Table 6. When these metrics given in Table 6 are investigated, it is easily seen that the convergence performance of pIPA is more robust than the convergence performance of IPA. Even though the Sr metrics of both pIPA and IPA are equal to 100% for $f_{1}$, $f_{6}$, $f_{8}$, $f_{10}$ and $f_{11}$ functions, the Me metric of pIPA is better than the Me metric of IPA. For all the remaining benchmark functions, pIPA outperforms the standard implementation of the IPA by considering the convergence performance measured in terms of Sr and Me. The Figure 2 given should also be viewed to investigate the changes in the convergence curves of the pIPA with the varying prc values.

The final comparison between pIPA and other meta-heuristics for 100-dimensional problems was carried out to decide whether the result of pIPA is enough to generate a statistical difference in favor of pIPA or not using the Wilcoxon signed rank test with the significance level equal to 0.05. If the significance level shown as ρ is less than 0.05, it is said that the difference between the two algorithms is statistically significant in favor of one of them. Otherwise, the results obtained by the algorithms are not enough to decide which one is statistically significant. The statistical test results related to the pIPA and its competitors were given in Table 7. In Table 7, R+ and R− show the sum of positive ranks and the sum of negative ranks, respectively. Also, the *Z* corresponds to the standardized test statistic. The results given in Table 7 show that the statistical difference between pIPA and MFO, PSO, GSA, BA, FPA, SMS, FA, or GA is in favor of pIPA. Only the decision about whether a statistical difference between pIPA and IPA exists or not cannot be made from the current results of the algorithms.

The qualities of the final solutions, convergence performance, and statistical test results of the pIPA for 100-dimensional problems gave strong evidence of its capabilities. However, its capabilities should also be analyzed with another scenario in which population size, dimensionalities of the problems, and termination criteria are changed. For this purpose, the benchmark functions given in Table 1 were solved by setting the population size of the pIPA to 100 and number of parameters to 200 [25,51]. The maximum evaluation number was taken equal to 500,000 [25,51]. Nine positive integers including 30, 35, 40, 50, 60, 70, 80, 90 and 95 were assigned to the prc and pIPA was tested 30 times with random seeds for each problem instance and prc combination. The objective function values of the best solutions found for each of 30 runs were averaged and reported in Table 8 with the related standard deviations.

When the results given in Table 8 are investigated, it is seen that the change trend of the pIPA with the different prc for 200-dimensional problems is similar to the change trend of the pIPA with the different prc for 100-dimensional problems. The pIPA obtains the global minimum solutions with the different values assigned to the prc for the $f_{1}$, $f_{6}$, $f_{9}$, and $f_{11}$ functions. Moreover, it finds almost the same mean best objective function values for the $f_{5}$ function with all nine different values of the prc as in the previous experimental settings. For $f_{2}$, $f_{9}$, and $f_{12}$ functions, pIPA obtains better or slightly changed solutions when the value assigned to the prc increases from 30 to 80 or even 90. Similar generalization can also be made for the $f_{3}$ and $f_{4}$, $f_{10}$ functions by considering the prc increasing from 30 to 40 and $f_{7}$ function by considering the prc increasing from 60 to 90. However, it should be noted that more robust solutions for the $f_{7}$ function can be obtained with the prc less than 40.

The changes in the average number of donors and receivers of the pIPA for 200-dimensional benchmark functions can be examined with Table 9. As seen from Table 9, pIPA tries to adjust the number of donors and receivers for the $f_{1}$, $f_{4}$, $f_{6}$, $f_{9}$, $f_{10}$ and $f_{11}$ functions while the number of donors and receivers remains unchanged for the other functions. Choosing the value of the prc relatively close to its upper or lower bound decreases the number of possible donors or receivers. However, the donor–receiver selection strategy of the pIPA can increase the number of donors compared to the number of donors determined with the value of the prc, if the objective function values of the qualified individuals are relatively close to each other or same. Otherwise, the number of donors and receivers is simply calculated using the assigned value to the prc.

The results of the pIPA for 200-dimensional problems should be validated with the comparison to the results of other meta-heuristics obtained under the same conditions. For this purpose, pIPA was compared with the standard implementation of the IPA [51], ALO [25], PSO [55], SMS [44], BA [19], FPA [20], CS [17], FA [18] and GA [6]. Although population sizes of the tested algorithms were equal to 100, the maximum evaluation number was set to 500,000. The prc of the pIPA was 90 for $f_{1}$, $f_{2}$, $f_{5}$, $f_{6}$, $f_{8}$ and $f_{12}$. Also, it was determined as 40 for the $f_{3}$, $f_{4}$, $f_{7}$, $f_{9}$, $f_{10}$ and $f_{11}$. When the mean best objective function values and standard deviations of the algorithms in Table 10 are controlled, it is seen that pIPA outperforms other tested algorithms or obtains the same mean best objective function values for ten of 12 benchmark functions. Although pIPA lags behind the ALO for the $f_{5}$ function and the IPA for the $f_{8}$ function, it becomes the second-best algorithm among other competitors for these functions and proves its superiority with the average rank equal to 1.1667.

The comparison between pIPA and other meta-heuristics for classical benchmark problems was completed by the results of the Wilcoxon signed rank test with the significance level 0.05. From the test results given in Table 11, it is understood that the solutions obtained by the pIPA for 200-dimensional problems are strong enough to generate a statistical difference in favor of the pIPA. Although the ρ value is found equal to 0.0022 for the statistical comparison between the pIPA and PSO, SMS, BA, FPA, CS, FA, or GA and proves that the difference is in favor of pIPA, the ρ value is found equal to 0.0151 for the statistical comparison between pIPA and ALO and 0.0285 for the statistical comparison between pIPA and IPA. The results found by the IPA for the $f_{8}$ function and ALO for the $f_{5}$ function cause a slight change in the ρ values. However, it is still less than 0.05, and validates the comparative performance of the pIPA.

### 4.2. Solving CEC 2015 Benchmark Problems with pIPA

**Figure 3:**
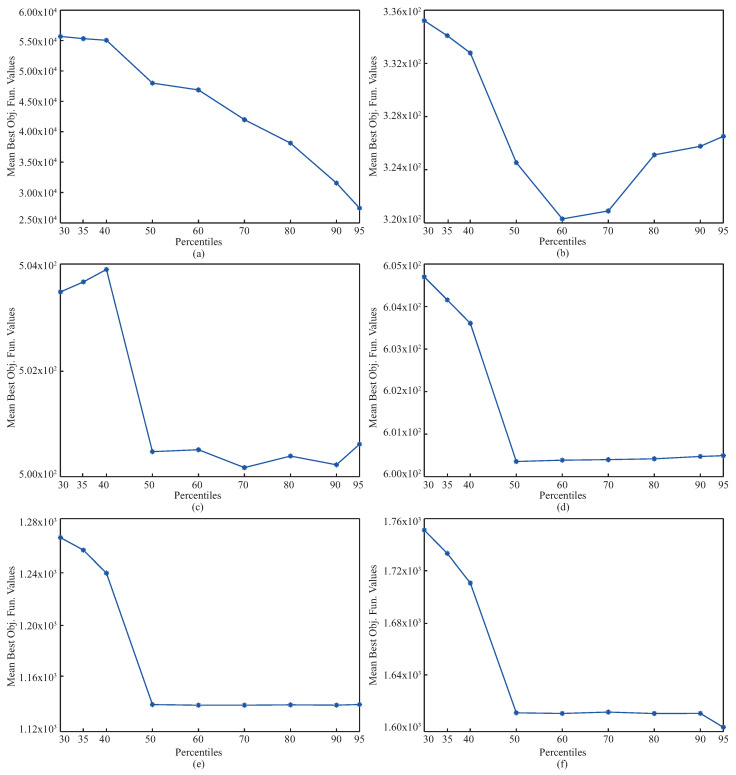
Changes in pIPA with different prc values for $f_{2}$ (**a**), $f_{3}$ (**b**), $f_{4}$ (**c**), $f_{5}$ (**d**), $f_{9}$ (**e**) and $f_{10}$ (**f**) functions.

The complexities of the benchmark problems can be increased using operations related to shifting, rotation, hybridization, and composition. To investigate the performance of pIPA on solving these kinds of problems, ten different 30-dimensional problems introduced at CEC 2015 were chosen, and their names, base functions, and global minimums are listed in Table 12 [61]. The lower and upper bounds of these functions were equal to −100 and +100 [61]. Although the $f_{1}$ and $f_{2}$ functions in Table 12 are rotated, $f_{3}$, $f_{4}$, $f_{5}$, $f_{6}$, $f_{7}$ and $f_{8}$ functions are both shifted and rotated [61]. Moreover, while the $f_{9}$ is a hybrid function generated by four base functions, the $f_{10}$ is a compositional function joining three base functions [61]. When solving the problems given in Table 12, the population size of the pIPA was set to 100, and the maximum evaluation number was taken equal to 100,000 [37]. Nine different values including 30, 35, 40, 50, 60, 70, 80, 90 and 95 were assigned to prc and pIPA was tested 30 times with random seeds for each problem and prc combination. The objective function values of the best solutions found by each of 30 runs were averaged and reported in Table 13 with the related standard deviations. The results given in Table 13 guide us to interpret the change trend of the pIPA with the varied prc values. For the $f_{1}$ and $f_{2}$ functions, the objective function values of the obtained solutions by pIPA grow better with the prc increasing from 30 to 90 or 95. Similar generalizations can be made for the remaining functions except $f_{5}$. Although the increasing values of prc from 30 to 60 or 70 improves the qualities of the solutions found by pIPA for $f_{3}$, $f_{7}$, $f_{9}$ and $f_{10}$ functions, the qualities of the solutions found by pIPA grow better with the prc increasing from 30 to 50. Only for the $f_{5}$ function do the values assigned to the prc not cause a significant change in the solution qualities of the pIPA. Figure 3 should also be viewed to analyze the effect of the prc on the performance of the pIPA.

For validating the qualities of the solutions found by the pIPA, its mean best objective function values and standard deviations are compared with the mean best objective function values and standard deviations belonging to the IPA [51], SOA [37], SHO [35], GWO [23], PSO [55], MFO [24], MVO [27], SCA [26], GSA [41], GA [6] and DE [56] as in Table 14. To ensure that the comparison is made under the same conditions, the population size and maximum evaluation number were set to 100 and 100,000 for all algorithms [37]. The prc value of the pIPA was taken equal to 50 for $f_{5}$ and $f_{6}$ functions, 60 for $f_{3}$ and $f_{9}$ functions, 70 for $f_{4}$ function, 80 for $f_{7}$ function, 90 for $f_{1}$ and $f_{8}$ functions, 95 for $f_{2}$ and $f_{10}$ functions. The NoR and NoD parameters of the IPA were set to 1. The results given in Table 14 showed that the pIPA and IPA outperform SOA, SHO, GWO, PSO, MFO, MVO, SCA, GSA, GA and DE for the $f_{4}$, $f_{5}$, $f_{6}$, $f_{8}$, $f_{9}$ and $f_{10}$ functions. Although all the tested algorithms find the same mean best objective function values for the $f_{3}$ function, SOA outperforms tested algorithms for the $f_{1}$ function, and GSA outperforms tested algorithms for the $f_{2}$ function.

### 4.3. Solving Noise Minimization Problem with pIPA

The volume, velocity, variety, and veracity properties of the data moved the difficulties of data-dependent optimization problems into another stage [62,63]. One of the data-dependent optimization problems has recently been introduced by Abbass et al., and a special competition has been organized at CEC 2015 with the name BigOpt [64]. The real-world optimization problem introduced by Abbass et al. mainly focuses on minimizing the measurement noise of the electro-encephalography (EEG) signals [65,66]. They stored 0.5 megabit of binary formatted data and 20 kilobyte of text formatted data per second and organized them for providing different problem instances. If the measurement of the EEG signal is extended to a period of time, a unique problem instance per second by neglecting the time spent for the storage and preparation will be encountered. Assume that *X* and *S* are two different matrices of size N×M. Although *N* corresponds to the number of time series belonging to the EEG signal, *M* is used on behalf of the number of elements for a time series. In addition to the *X* and *S* matrices, there is a square transformation matrix *A* of size N×N, and it relates *S* matrix to the *X* matrix as described in Equation (7) [64]. If the *S* matrix is matched with the EEG signal containing *N* time series with *M* samples in each series, the noise-free part of the *S* showed by $S_{1}$ and the noise part of the *S* showed by $S_{2}$ must be obtained and used with the *A* matrix for finding *X* as in Equation (8) [65,66]. Even though the relationship between *S*, $S_{1}$ and $S_{2}$ matrices is straightforward, a simple method splitting the *S* matrix into $S_{1}$ and $S_{2}$ matrices cannot be found easily. By considering the difficulty of splitting the *S* matrix, Abbass et al. decided to guide the Pearson Correlation Coefficients showed as *C* in Equation (9) [66]. In Equation (9), $covar(X,A\times S_{1})$ is the covariance matrix and var(X) and $var(A\times S_{1})$ are variance matrices, respectively.

(7) X=A×S

(8) $$X=A\times \left(S_{1}+S_{2}\right)=A\times S_{1}+A\times S_{2}$$

(9) $$C=\frac{covar(X,A\times S_{1})}{var\left(X\right)\times var\left(A\times S_{1}\right)}$$

Abbass et al. also stated that the diagonal and off-diagonal elements of the *C* matrix have important information about the appropriateness of the $S_{1}$ matrix and can be referenced for splitting the original *S* matrix [65,66]. Although the $S_{1}$ matrix is obtained from the *S* matrix, the diagonal elements of the *C* should be maximized, and other elements should be minimized by considering the upper and lower bounds. To understand how the calculated *C* matrix for a guessed $S_{1}$ satisfies the mentioned properties about the diagonal and off-diagonal elements, Equation (10) is utilized [65,66].

(10) $$f_{1}=\frac{1}{\left(N^{2}-N\right)}\sum_{i\neq j}{\left(C_{ij}\right)}^{2}+\frac{1}{N}\sum_{i}{\left(1-C_{ii}\right)}^{2}$$

Another important situation that should be controlled when the $S_{1}$ matrix is tried to be determined is its similarity with the original *S* matrix. Because the $S_{1}$ matrix represents the noise-free part of the original *S* matrix, the difference between *S* and $S_{1}$ matrices should be minimized. For measuring the difference between *S* and $S_{1}$ matrices, Equation (11) can be used [65,66]. As easily seen from Equation (11), the $S_{1}$ matrix should be chosen relatively close to the *S* matrix for representing the properties of the EEG signal. When the $S_{1}$ matrix is tried to be found by guiding the minimization of the sum of $f_{1}$ and $f_{2}$, an optimization problem can be introduced. For analyzing the performance of the solving techniques on the mentioned optimization problem, different instances named D4, D4N, D12, and D12N were introduced by Abbass et al. and required *X*, *A*, and *S* matrices for each instance were reported [65,66]. The D4 and D12 instances have four and 12 time series with length 256. Similar to the D4 and D12 instances, D4N and D12N instances also have four and 12 time series with length 256. However, these problem instances are changed slightly with the additional noise components.

(11) $$f_{2}=\frac{1}{N\times M}\sum_{ij}{\left(S_{ij}-S1_{ij}\right)}^{2}$$

The pIPA was tested for solving the D4, D4N, D12, and D12N problem instances. The population size of pIPA was set to 50 [51]. Nine different values including 30, 35, 40, 50, 60, 70, 80, 90 and 95 were assigned to the prc. For each combination of problem instance and prc, pIPA was tested 30 times with random seeds by setting the maximum evaluation number to 10,000 [51]. The mean best objective function values and standard deviation of each test scenario were recorded and presented in Table 15. The results given in Table 15 showed that mean best objective function values of pIPA decrease with the increasing value of the prc from 30 to 80 for D4, D4N, and D12 problem instances and increasing value of the prc from 30 to 90 for D12N problem instance. Although the appropriate value of the prc parameter is 80 for D4, D4N, and D12 problem instances by considering the mean best objective function values, the appropriate value of the prc parameter is 90 for D12N problem instance by considering the mean best objective function values.

The results obtained by the pIPA for noise minimization problem were compared with the results of IPA [51], GA [6], PSO [55], DE [56], ABC [57], GSA [41], MFO [24], SCA [26], SSA [28] and HHO [29]-based techniques. To guarantee that the comparison is made under equal conditions, the population or colony size of the algorithms was set to 50, and the maximum evaluation number was taken as 10,000 [51]. The prc parameter of pIPA was set to 80 for the D4, D4N, and D12 problem instances and 90 for the D12N problem instance. The NoD and NoR parameters of the standard IPA were equal to 4 and 8, respectively [51]. For the GA, the crossover rate was 0.95, and the mutation rate was 0.001. The inertia weight of PSO achieved its value between 0.2 and 0.9, and both $c_{1}$ and $c_{2}$ acceleration coefficients were set to 2. Although the scaling factor of DE achieved its value randomly between 0.2 and 0.8, the crossover rate was taken equal to 0.9. The limit parameter of ABC was set to the half of PS×D where *D* was equal to 1024 for D4 and D4N and 3072 for D12 and D12N. The calculation of the logarithmic spiral was completed by setting the *b* constant to 1 for MFO. Assuming that *l* and *L* are current and maximum iteration numbers, the $c_{1}$ coefficient of SSA was calculated as $2e^{-(16l^{2}/L^{2})}$. When the best, mean best objective function values and standard deviation over 30 independent runs given in Table 16 are controlled, it is seen that pIPA removes artifacts or noises more robustly compared to the other tested algorithms for all four problem instances. The percentile-based donor–receiver selection strategy that already proved its efficiency in solving classical benchmark problems also contributes to the performance of the algorithm, and more robust $S_{1}$ matrices are obtained.

As stated earlier, if the measurement of the EEG signal is extended to a period of time, a unique problem instance per second will be encountered, and algorithms should generate their solutions within a second to successfully handle the subsequent instance. To decide whether the pIPA and some of its competitors, including IPA and ABC, can produce their solutions within a second or not using the existing test configuration, the average execution times in terms of seconds were calculated and then presented in Table 17. The pIPA, IPA, and ABC were coded in C programming language. Also, all experiments were carried out on a PC equipped with a single-core 1.33 Ghz processor. The results of Table 17 help to state that neither pIPA nor IPA is capable of filtering EEG instances within a second. Especially for the problem instances with 12 time series, parallelization of the algorithms is seen as a necessity for processing the ongoing measurements.

The comparative studies between meta-heuristics should be supported with the appropriate statistical tests. By considering this requirement, the Wilcoxon signed rank test with the significance level 0.05 was used again for determining whether a statistical difference between pIPA and other tested meta-heuristics exists or not. The test results given in Table 18 represent that the contribution of the newly proposed selection mechanism is enough to generate a statistical difference in favor of pIPA. The results also help to state that pIPA outperforms its competitors in almost all the 30 different runs related to the D4, D4N, D12, and D12N instances when the calculated ρ values are considered.

### 4.4. Solving Path Planning Problem with pIPA

The operational success and safety of a UAV or UCAV are directly related to the path or flight route on the battlefield equipped by using sophisticated anti-air weapon systems, radars, missiles, and artilleries [67]. The path or route determined on the task region for a UAV or UCAV should minimize the probability of being shot down and fuel consumption [67]. By considering these objectives, Xu et al. proposed a mathematical model describing how a path from the start point $P_{s}=\left(x_{s},y_{s}\right)$ to the target point $P_{t}=\left(x_{t},y_{t}\right)$ can be found optimally [67]. The model described by Xu et al. first divides the line between $P_{s}$ to $P_{t}$ equally into (D+1) segments using *D* different segmentation points. Each segmentation point is intersected vertically by a line, and a set of lines showed as $L=\{L_{1},L_{2},\ldots ,L_{D}\}$ is generated [67]. If a point is found on each line in the set *L* and then these points are connected one by one, a single path from the start point $P_{s}$ to target point $P_{t}$ can be described as a set of points showed as $P=\{P_{s},P_{1},P_{2},\ldots ,P_{D-1},P_{D},P_{t}\}$.

The search operations of points in the set *P* except the $P_{s}$ and $P_{t}$ can be further simplified by appropriately transforming the current coordinate system. If the current coordinate system is transformed in a manner that the line between the $P_{s}$ and $P_{t}$ corresponds to the horizontal axis in the new coordinate system, each point tried to be determined is represented only single parameter [67]. For transforming the $\left(x_{k},y_{k}\right)$ point of the original coordinate system into the suitable point of the new coordinate system, Equation (12) is employed [67]. In Equation (12), θ is the rotation angle between the *x*-axis of the original coordinate system and the line between $P_{s}$ and $P_{t}$ and calculated as $arctan(\left(y_{t}-y_{s}\right)/\left(x_{t}-x_{s}\right))$ [67].

(12) $$\left[\begin{array}{c} x_{k}^{{}^{\prime }}\\ y_{k}^{{}^{\prime }} \end{array}\right]=\left[\begin{array}{cc} cos(\theta ) & sin(\theta )\\ -sin(\theta ) & cos(\theta ) \end{array}\right]\times \left[\begin{array}{c} x_{k}-x_{s}\\ y_{k}-y_{s} \end{array}\right]$$

When the required points are determined, the suitability of the path generated using these points can be estimated with Equation (13) [67]. In Equation (13), *J* corresponds to the sum of costs related to the enemy threats and fuel consumption weighted using the λ and (1−λ), respectively. Also, while the $w_{t}$ represents the cost of enemy threats changing with the length of path abbreviated as *l*, $w_{f}$ is used on behalf of the cost of fuel consumption changing with the *l* [67].

(13) $$J=\lambda \int_{0}^{l}w_{t}dl+\left(1-\lambda \right)\int_{0}^{l}w_{f}dl$$

Even though the equation used for determining the suitability of the path is relatively simple, it can be further purified by replacing the integral calculations with their appropriate approximations [67]. For this purpose, the $w_{f}$ is first taken equal to 1, and the integral calculation about the cost of fuel consumption becomes directly proportional to the length of the path [67]. Second, the integral calculation about the cost of enemy threats is changed with an approximation in which the cost of threats is determined for each segment of the path. Assume that $L_{ij}$ is the segment between the segmentation points *i* and *j*. In addition to this, $L_{ij}$ is divided equally into ten sub-segments, and the first, third, fifth, seventh, and ninth sub-segmentation points are selected. For the cost of all $N_{t}$ threats related to the $L_{ij}$, the summation described in Equation (14) is utilized [67]. Given that $t_{k}$ is the degree of the threat *k*, if the segment of length $L_{ij}$ is within the effect range of the threat *k*, the cost of threat *k* showed as cost(k,m) for the sub-segmentation point *m* is found equal to $t_{k}/d\left(k,m\right)$ where d(k,m) corresponds to the Euclidean distance between the center of threat *k* and sub-segmentation point *m*.

(14) $$J_{t,(ij)}=\frac{L_{ij}}{5}\sum_{k=1}^{N_{t}}\sum_{m}^{\left\{1,3,5,7,9\right\}}cost\left(k,m\right)$$

**Figure 4:**
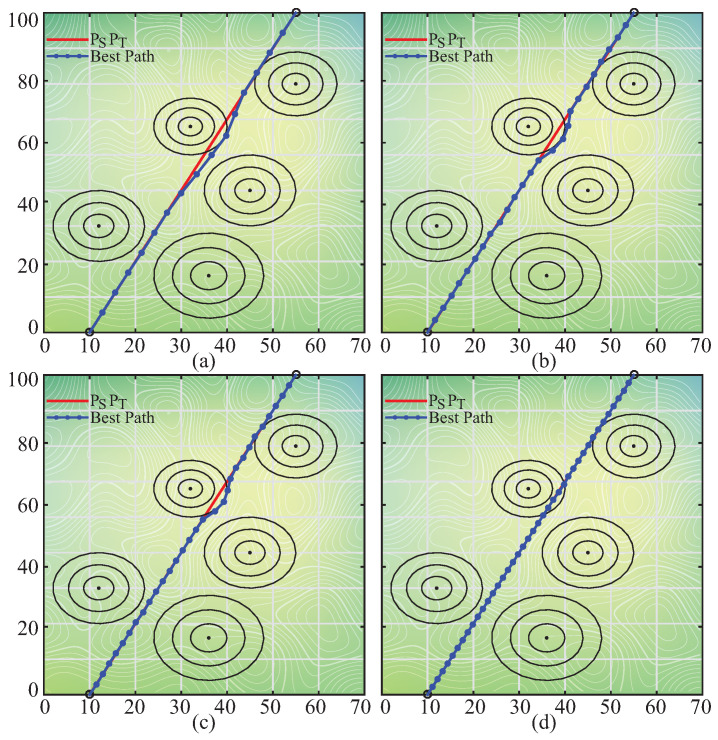
The best paths found by pIPA for *D* equal to 15 (**a**), 25 (**b**), 30 (**c**) and 40 (**d**).

For investigating the performance of the pIPA on the path planning problem, the battlefield whose details are given in Table 19 was used [58,59]. The number of segmentation points or *D* was taken equal to 5, 10, 15, 20, 25, 30, 35 and 40 [58,59]. The value of the λ coefficient was determined as 0.5 [58,59]. The population size of the pIPA and maximum evaluation number were set to 30 and 6000 [58,59]. Six different values including 50, 60, 70, 80, 90 and 95 were assigned to the prc sequentially. The pIPA was tested 100 times with random seeds for each combination about *D* and prc. The best, worst, mean best objective function values and standard deviations of 100 runs were recorded and presented in Table 20. The results presented in Table 20 state that the prc value should be chosen between 60 and 70. Although the pIPA obtains more suitable paths for the UCAV on the battlefield with *D* equal to 15, 20, 25, 30, and 35 by setting the prc to 60, the appropriate value of the prc for the remaining battlefield configurations is equal to 70. The best paths found by the pIPA with prc set to 60 for different cases can be visualized as in Figure 4.

The qualities of the paths found by the pIPA should be compared with the qualities of the paths obtained by different meta-heuristics. For this purpose, the best, worst, mean best objective function values and standard deviations found by the pIPA with prc equal to 60 were compared to the corresponding results of the IPA [68] with NoD and NoR equal to 1, ABC [68] with limit equal to 100, BA [58], BAM [58], ACO [59], BBO [59], DE [59], ES [59], FA [60], GA [59], MFA [60], PBIL [59], PSO [59], SGA [59] and PGSO [59]-based UCAV path planners. To guarantee that the results were obtained under the same conditions, the population or colony size of the mentioned algorithms was set to 30. Each algorithm was executed 100 times by taking a maximum evaluation number equal to 6000, and their results were summarized in Table 21. The results given in Table 21 showed that the pIPA is the best path planner with the average rank calculated as 1.500 among all 16 meta-heuristics when the mean best objective function values are considered. It outperforms other tested algorithms or shares the first rank for the battlefield with *D* set to 5, 15, 30, 35, and 40. Moreover, the paths found by the pIPA for the battlefield with *D* set to 20 and 25 are in the second rank by considering the mean best objective function values. Even though the path obtained by the pIPA for the battlefield with *D* set to 10 lags slightly behind its competitors, it is still in the third rank and produces a better path compared to 13 different algorithms.

The contribution of the percentile-based selection strategy on the convergence performance can be guessed by referencing the paths and their qualities belonging to the pIPA. However, unique properties of the UCAV path planning problem require a further control for Sr and Me metrics. For this purpose, the Sr and Me values of the pIPA, IPA, and ABC were calculated by adjusting the threshold to 55 and given in Table 22. When the Sr and Me metrics of Table 22 are investigated, it can be seen that pIPA with prc equal to 50 or 60 obtains paths whose qualities are equal to the determined threshold or better for all eight battlefield configurations at each of 100 different runs. Moreover, the pIPA with prc set to 70, 80, or 90 still protects its stability and converges more quickly compared to the IPA and ABC for most of the test cases. Although the pIPA with prc set to 60 converges 1.600 times faster compared to IPA for the battlefield with *D* equal to 25, it converges 1.513, 1.393 and 1.450 times faster compared to IPA for the battlefield with *D* equal to 30, 35 and 40.

The comparative studies between pIPA and other techniques for the UCAV path planning problem were concluded by controlling the results of the Wilcoxon signed rank test with the significance level of 0.05. The test results were calculated using the best objective function values and then presented in Table 23. As easily seen from the test results, the difference between pIPA and IPA, ABC, BA, ACO, BBO, DE, ES, GA, PBIL, PSO, SGA, or PGSO is enough to generate a statistical difference in favor of the pIPA. Only the difference between the pIPA and BAM, FA, or MFA is not enough to state that there is a statistical significance in favor of the pIPA. However, it should be noted that the ρ value calculated for the comparison between pIPA and BAM or FA is relatively close to 0.05 and supplies information about the qualities of the paths found by pIPA.

## 5. Results and Discussion

The standard implementation of the IPA determines the number of donors by assigning a constant to the NoD parameter and selects the best NoD individual or individuals from the population as donor or donors. Similarly, IPA determines the number of receivers by assigning a constant value to the NoR parameter and selects the worst NoR individual or individuals from the population as receiver or receivers. Even though the usage of NoD and NoR control parameters increases the flexibility of the IPA, deciding which values will be convenient for these control parameters and guessing the interaction between them are difficult. Moreover, solving some optimization problems with IPA can require adaptive adjustment for the number of donors and receivers.

The main idea lying behind the newly introduced donor–receiver selection strategy is providing an improved mechanism that both simplifies the initialization of the IPA and allows the algorithm to determine the number of donors and receivers adaptively. When the pIPA improves the qualities of the individuals in the population, it tries to extend the set of possible donors, as easily seen from Table 3 and Table 9 presenting the change trends of the number of donors and receivers for 100 and 200-dimensional benchmark problems even though the prc remains unchanged. If the number of possible donors is increased by the pIPA, the chance of plasma transfer to a receiver from a different donor is also increased. Moreover, if the pIPA decides to increase the number of donors, the number of receivers is decreased simultaneously, and more critical receivers, i.e., poor solutions, have a chance of improving their qualities. The pIPA can also decrease the number of donors. If the number of donors is decreased, the number of receivers is increased simultaneously. Because some donor candidates with relatively low qualities are discarded from the set of possible donors, receivers have a chance of treatment with the more qualified or better donors. Finally, if the pIPA decides that there is no receiver in the current infection cycle, any treatment operations are not carried out, and the infection continues to spread between the individuals of the population and exploration characteristic of the search becomes more dominant.

As an expected result of the properties related to the percentile-based donor–receiver selection strategy, the pIPA outperformed standard implementation of IPA and other meta-heuristics for the vast majority of the tested numerical and complex optimization problems. Although the contribution of the proposed model on the qualities of the final solutions and convergence performance becomes more apparent for the 100 and 200-dimensional classical problems, pIPA loses its advantageous sides for some of the CEC 2015 problems. The most powerful side of the pIPA is adjusting the number of donors and receivers by considering the qualities of the individuals in the population. Although the value assigned to prc remains the same until the end of execution, pIPA utilizes the special property of the percentile calculation and changes the sets of possible donors and receivers. However, some problems introduced at CEC 2015 are generated by hybridization or composition of two or more basic functions. Because of this main reason, while the assigned value to the prc and set of possible donors and receivers are appropriate for a basic function, another participating function requires a more subtle number of donors and receivers for the plasma treatment as in the standard IPA.

When the results obtained by the pIPA for the complex engineering problems are investigated, the positive contribution of the newly proposed technique on the quality of the final solution and convergence speed is understood again. The EEG noise minimization is a big-data optimization problem and requires processing 1024 parameters at a second for D4 and D4N instances and 3072 parameters at a second for D12 and D12N instances. The difficulties of the problem stemmed from the high dimensionality and conflicting objectives, claiming a more sensitive search within the promising solutions. In the pIPA, the required sensitive search by considering the neighborhood of the promising solutions can be satisfied by decreasing the number of donors or assigning an initial value of the prc big enough. Another tested engineering problem, also called path planning, slightly differs from other problems when the number of segmentation points is considered. If the number of segmentation points or *D* increases, the possibility of finding a segmentation point within the circles representing the enemy air defense systems is also increased intrinsically. Moreover, it should be noted that even though the *D* is relatively small, some segmentation points can still be relatively close to the centers of enemy threats. Because of the specific properties of the UCAV path planning problem, the algorithms being tested should be capable of escaping local optimal solutions more quickly. In the pIPA, the exploration or exploitation dominant operations are tried to be managed adaptively. Although the value of the prc parameter is set to a constant such as 50, 60, 70 and even 80, the pIPA is capable of finding a balance between exploration and exploitation dominant operations and more safe and robust paths are obtained compared to the standard IPA and other meta-heuristics. However, if the prc value is not determined appropriately and the maximum number of evaluations is not selected relatively high, it should be noted that the pIPA can consume a substantial amount of function evaluations for accessing the required balance and terminates without obtaining promising solutions.

## 6. Conclusions

In this study, the donor–receiver selection strategy of the immune plasma algorithm (IP algorithm or IPA) was modified by guiding a statistical measure known as the percentile and then an improved IPA variant called the percentile IPA (pIPA) was introduced. To analyze how the newly introduced donor–receiver selection strategy contributes to the solving capabilities of the pIPA, a set of experiments was carried out. In the first and second parts of the experimental studies, 22 numerical benchmark problems were solved with the pIPA by assigning different values to its control parameters, and the obtained results were compared to the classical and state-of-art meta-heuristics including IPA, PSO, GSA, CS, BA, FPA, SMS, FA, GA, MFO, ALO, SOA, SHO, GWO, MVO, SCA and DE. The third part of the experimental studies was devoted to the investigations about the pIPA using a big-data optimization problem requiring noise minimization in the EEG signals, and pIPA was compared to the IPA, GA, PSO, DE, ABC, GSA, MFO, SCA, SSA, and HHO-based techniques. Finally, in the fourth part of the experimental studies, pIPA was used to find an optimal flight path for a UCAV, and its results were compared to the results of the IPA, ABC, BA, BAM, ACO, BBO, DE, ES, FA, GA, MFA, PBIL, PSO, SGA and PGSO-based planners.

The comparative studies showed that the proposed strategy contributes to the convergence performance and qualities of the final solutions obtained by the pIPA, and it performs better than other tested algorithms for most of the benchmark cases. Adjusting both the possible donors and receivers using only one parameter called prc in the pIPA removes the necessity of NoD and NoR parameters and reduces the total number of control parameters defined for the standard IPA. Moreover, even though the value assigned to the prc is a constant, the number of donors and receivers can vary from one infection cycle to another because of the definition of the percentile. The promising results of the experimental studies also informed that future works about the IPA can focus on developing different donor–receiver selection approaches, adaptive adjustment strategies for the number of donors–receivers, and their applications in various numerical or combinatorial problems. In addition to these future research proposals, the IPA and pIPA can be extended with the usage of multiple populations. Each has its own donor–receiver selection and treatment mechanisms or parallelization that divides a single population into simultaneously executing small populations.

## Figures and Tables

**Table 1:** Classical benchmark functions used in experiments.

Name	Range	Formulation
Sphere	[−100, 100]	$f_{1}\left(\vec{x}\right)=\sum_{i=1}^{D}\left(x_{i}^{2}\right)$
Schwefel2.22	[−10, 10]	$f_{2}\left(\vec{x}\right)=\sum_{i=1}^{D}|x_{i}|+\prod_{i=1}^{D}\left|x_{i}\right|$
Schwefel1.2	[−100, 100]	$f_{3}\left(\vec{x}\right)=\sum_{i=1}^{D}\sum_{j=1}^{i}x_{j}$
Schwefel2.21	[−100, 100]	$f_{4}\left(\vec{x}\right)=max_{i}\left(\right|x_{i}\left|,\;1\leq i\leq D\right)$
Rosenbrock	[−30, 30]	$f_{5}\left(\vec{x}\right)=\sum_{i=1}^{D-1}{\left(100(x_{i+1}-x_{i}^{2}\right)}^{2}+{\left(x_{i}-1\right)}^{2})$
Step	[−100, 100]	$f_{6}\left(\vec{x}\right)=\sum_{i=1}^{D}{\left(\lfloor x_{i}+0.5\rfloor \right)}^{2}$
Random	[−1.28, 1.28]	$f_{7}\left(\vec{x}\right)=\sum_{i=1}^{D}\left(ix_{i}^{4}\right)+random\left[0,1\right)$
Schwefel	[−500, 500]	$f_{8}\left(\vec{x}\right)=\sum_{i=1}^{D}\left(-x_{i}sin\left(\sqrt{\left|x_{i}\right|}\right)\right)$
Rastrigin	[−5.12, 5.12]	$f_{9}\left(\vec{x}\right)=\sum_{i=1}^{D}\left(x_{i}^{2}-10cos\left(2\pi x_{i}\right)+10\right)$
Ackley	[−32, 32]	$f_{10}\left(\vec{x}\right)=20+e-20exp\left(-0.2\sqrt{\frac{1}{D}\sum_{i=1}^{D}x_{i}^{2}}\right)-exp\left(\frac{1}{D}\sum_{i=1}^{D}cos\left(2\pi x_{i}\right)\right)$
Griewank	[−600, 600]	$f_{11}\left(\vec{x}\right)=\frac{1}{4000}\left(\sum_{i=1}^{D}x_{i}^{2},\right)-\left(\prod_{i=1}^{D}cos\left(\frac{x_{i}}{\sqrt{i}}\right)\right)+1$
Penalized	[−50, 50]	$\begin{array}{c} \begin{array}{c} f_{12}\left(\vec{x}\right)=\frac{\pi }{D}\left(10sin^{2}\left(\pi y_{i}\right)+\left(\sum_{i=1}^{D}\left(y_{i}{-1)}^{2}(1+10sin^{2}(\pi y_{i+1}\right)\right)\right)\\ +\\ \sum_{i=1}^{D}u\left(x_{i},10,100,4\right) \end{array}\\ \begin{array}{cc} u\left(x_{i},a,k,m\right)=\left\{\begin{array}{cc} k{\left(x_{i}-a\right)}^{m}, & x_{i}>a\\ 0, & -a\leq x_{i}\leq a\\ k{\left(-x_{i}-a\right)}^{m}, & x_{i} \end{array}\right\} & y_{i}=1+\frac{1}{4}\left(x_{i}+1\right) \end{array} \end{array}$

**Table 2:** Results of pIPA with different prc values for 100-dimensional problems.

Fn.		prc								
		30	35	40	50	60	70	80	90	95
$f_{1}$	Mean	$5.1456\times 10^{-26}$	$1.3752\times 10^{-30}$	$2.0262\times 10^{-34}$	$2.2410\times 10^{-36}$	$1.2098\times 10^{-43}$	$5.8013\times 10^{-46}$	$5.1764\times 10^{-51}$	$4.6656\times 10^{-49}$	$5.0181\times 10^{-42}$
	Std.	$2.7982\times 10^{-25}$	$5.1533\times 10^{-30}$	$7.6024\times 10^{-34}$	$1.2270\times 10^{-35}$	$6.4963\times 10^{-43}$	$3.0141\times 10^{-45}$	$1.5408\times 10^{-50}$	$8.7556\times 10^{-49}$	$1.5187\times 10^{-41}$
$f_{2}$	Mean	$1.2335\times 10^{-15}$	$1.3169\times 10^{-17}$	$2.0901\times 10^{-21}$	$4.9870\times 10^{-26}$	$7.2877\times 10^{-29}$	$6.4561\times 10^{-31}$	$4.8992\times 10^{-33}$	$2.0915\times 10^{-35}$	$3.1866\times 10^{-30}$
	Std.	$2.7063\times 10^{-15}$	$3.4440\times 10^{-17}$	$4.9966\times 10^{-21}$	$1.1231\times 10^{-25}$	$9.9676\times 10^{-29}$	$1.5124\times 10^{-30}$	$1.7674\times 10^{-32}$	$3.9040\times 10^{-35}$	$1.2116\times 10^{-29}$
$f_{3}$	Mean	$3.1633\times 10^{-23}$	$8.5720\times 10^{-22}$	$9.3947\times 10^{-30}$	$1.0063\times 10^{-4}$	$6.3883\times 10^{2}$	$5.7721\times 10^{3}$	$1.1202\times 10^{4}$	$2.6989\times 10^{4}$	$5.6676\times 10^{4}$
	Std.	$9.9721\times 10^{-23}$	$4.6942\times 10^{-21}$	$3.3014\times 10^{-29}$	$5.5042\times 10^{-4}$	$3.4990\times 10^{3}$	$1.5215\times 10^{4}$	$2.5396\times 10^{4}$	$2.9479\times 10^{4}$	$4.7540\times 10^{4}$
$f_{4}$	Mean	$3.8292\times 10^{0}$	$4.5361\times 10^{0}$	$5.3971\times 10^{0}$	$9.2311\times 10^{1}$	$9.1797\times 10^{1}$	$9.2891\times 10^{1}$	$9.1533\times 10^{1}$	$9.1888\times 10^{1}$	$9.1597\times 10^{1}$
	Std.	$4.2740\times 10^{0}$	$2.9681\times 10^{0}$	$5.0036\times 10^{0}$	$1.6587\times 10^{0}$	$2.0900\times 10^{0}$	$1.3709\times 10^{0}$	$1.7876\times 10^{0}$	$2.7794\times 10^{0}$	$2.6573\times 10^{0}$
$f_{5}$	Mean	$9.8948\times 10^{1}$	$9.8953\times 10^{1}$	$9.8920\times 10^{1}$	$9.8708\times 10^{1}$	$9.8531\times 10^{1}$	$9.8629\times 10^{1}$	$9.8675\times 10^{1}$	$9.8851\times 10^{1}$	$9.8845\times 10^{1}$
	Std.	$5.2147\times 10^{-2}$	$1.0042\times 10^{-2}$	$6.5905\times 10^{-2}$	$1.9575\times 10^{-1}$	$4.4030\times 10^{-1}$	$3.6026\times 10^{-1}$	$2.9301\times 10^{-1}$	$7.3941\times 10^{-2}$	$9.0345\times 10^{-2}$
$f_{6}$	Mean	$0.0000\times 10^{0}$	$0.0000\times 10^{0}$	$0.0000\times 10^{0}$	$0.0000\times 10^{0}$	$0.0000\times 10^{0}$	$0.0000\times 10^{0}$	$0.0000\times 10^{0}$	$0.0000\times 10^{0}$	$0.0000\times 10^{0}$
	Std.	$0.0000\times 10^{0}$	$0.0000\times 10^{0}$	$0.0000\times 10^{0}$	$0.0000\times 10^{0}$	$0.0000\times 10^{0}$	$0.0000\times 10^{0}$	$0.0000\times 10^{0}$	$0.0000\times 10^{0}$	$0.0000\times 10^{0}$
$f_{7}$	Mean	$1.5136\times 10^{-4}$	$1.7352\times 10^{-4}$	$1.1218\times 10^{-4}$	$1.3047\times 10^{-3}$	$1.0262\times 10^{-3}$	$9.7175\times 10^{-4}$	$1.2665\times 10^{-3}$	$1.3941\times 10^{-3}$	$1.5076\times 10^{-3}$
	Std.	$1.3928\times 10^{-4}$	$2.1698\times 10^{-4}$	$1.2010\times 10^{-4}$	$7.8270\times 10^{-4}$	$8.3336\times 10^{-4}$	$9.3030\times 10^{-4}$	$9.5681\times 10^{-4}$	$1.1079\times 10^{-3}$	$1.8053\times 10^{-3}$
$f_{8}$	Mean	$-1.4903\times 10^{4}$	$-1.4796\times 10^{4}$	$-1.4585\times 10^{4}$	$-2.1956\times 10^{4}$	$-2.2120\times 10^{4}$	$-2.2532\times 10^{4}$	$-2.3085\times 10^{4}$	$-2.3315\times 10^{4}$	$-2.3156\times 10^{4}$
	Std.	$4.5009\times 10^{2}$	$4.0984\times 10^{2}$	$3.3323\times 10^{2}$	$4.2788\times 10^{2}$	$4.7105\times 10^{2}$	$5.4506\times 10^{2}$	$4.3601\times 10^{2}$	$5.5671\times 10^{2}$	$7.7225\times 10^{2}$
$f_{9}$	Mean	$0.0000\times 10^{0}$	$0.0000\times 10^{0}$	$0.0000\times 10^{0}$	$0.0000\times 10^{0}$	$0.0000\times 10^{0}$	$0.0000\times 10^{0}$	$0.0000\times 10^{0}$	$0.0000\times 10^{0}$	$0.0000\times 10^{0}$
	Std.	$0.0000\times 10^{0}$	$0.0000\times 10^{0}$	$0.0000\times 10^{0}$	$0.0000\times 10^{0}$	$0.0000\times 10^{0}$	$0.0000\times 10^{0}$	$0.0000\times 10^{0}$	$0.0000\times 10^{0}$	$0.0000\times 10^{0}$
$f_{10}$	Mean	$8.4524\times 10^{-14}$	$3.5231\times 10^{-15}$	$1.2730\times 10^{-15}$	$1.9329\times 10^{1}$	$1.6653\times 10^{1}$	$1.7974\times 10^{1}$	$1.9291\times 10^{1}$	$1.9948\times 10^{1}$	$1.9292\times 10^{1}$
	Std.	$2.4061\times 10^{-13}$	$8.2258\times 10^{-15}$	$2.7492\times 10^{-15}$	$3.6507\times 10^{0}$	$7.5752\times 10^{0}$	$6.0939\times 10^{0}$	$3.6436\times 10^{0}$	$2.4914\times 10^{-2}$	$3.6437\times 10^{0}$
$f_{11}$	Mean	$0.0000\times 10^{0}$	$0.0000\times 10^{0}$	$0.0000\times 10^{0}$	$0.0000\times 10^{0}$	$0.0000\times 10^{0}$	$0.0000\times 10^{0}$	$0.0000\times 10^{0}$	$0.0000\times 10^{0}$	$0.0000\times 10^{0}$
	Std.	$0.0000\times 10^{0}$	$0.0000\times 10^{0}$	$0.0000\times 10^{0}$	$0.0000\times 10^{0}$	$0.0000\times 10^{0}$	$0.0000\times 10^{0}$	$0.0000\times 10^{0}$	$0.0000\times 10^{0}$	$0.0000\times 10^{0}$
$f_{12}$	Mean	$1.1622\times 10^{0}$	$1.1296\times 10^{0}$	$1.0991\times 10^{0}$	$7.2542\times 10^{-1}$	$7.5028\times 10^{-1}$	$7.4214\times 10^{-1}$	$7.3596\times 10^{-1}$	$7.9325\times 10^{-1}$	$8.3495\times 10^{-1}$
	Std.	$6.1114\times 10^{-2}$	$4.8698\times 10^{-2}$	$6.1272\times 10^{-2}$	$7.2941\times 10^{-2}$	$7.2955\times 10^{-2}$	$7.7846\times 10^{-2}$	$7.1764\times 10^{-2}$	$6.5839\times 10^{-2}$	$9.4424\times 10^{-2}$

**Table 3:** Average number of donors and receivers for 100-dimensional problems.

Fn.		prc								
		30	35	40	50	60	70	80	90	95
$f_{1}$	Dn.	21.000	20.000	18.000	15.000	12.000	9.000	6.000	3.000	2.000
	Rcv.	9.000	10.000	12.000	15.000	18.000	21.000	24.000	27.000	28.000
$f_{2}$	Don.	21.000	20.000	18.000	15.000	12.000	9.000	6.000	3.000	2.000
	Rcv.	9.000	10.000	12.000	15.000	18.000	21.000	24.000	27.000	28.000
$f_{3}$	Dn.	21.000	20.000	18.000	15.000	12.000	9.000	6.000	3.000	2.000
	Rcv.	9.000	10.000	12.000	15.000	18.000	21.000	24.000	27.000	28.000
$f_{4}$	Dn.	29.982	29.980	29.974	15.000	12.004	9.000	6.000	3.000	2.000
	Rcv.	0.018	0.020	0.026	15.000	17.996	21.000	24.000	27.000	28.000
$f_{5}$	Dn.	21.000	20.000	18.000	15.000	12.000	9.000	6.000	3.000	2.000
	Rcv.	9.000	10.000	12.000	15.000	18.000	21.000	24.000	27.000	28.000
$f_{6}$	Dn.	22.517	22.005	21.066	20.136	18.782	17.342	15.575	13.170	11.686
	Rcv.	7.483	7.995	8.934	9.864	11.218	12.658	14.425	16.830	18.314
$f_{7}$	Dn.	21.000	20.000	18.000	15.000	12.000	9.000	6.000	3.000	2.000
	Rcv.	9.000	10.000	12.000	15.000	18.000	21.000	24.000	27.000	28.000
$f_{8}$	Dn.	21.000	20.000	18.000	15.000	12.000	9.000	6.000	3.000	2.000
	Rcv.	9.000	10.000	12.000	15.000	18.000	21.000	24.000	27.000	28.000
$f_{9}$	Dn.	21.988	21.415	20.352	18.959	16.992	14.996	12.667	9.450	6.426
	Rcv.	8.012	8.585	9.648	11.041	13.008	15.004	17.333	20.550	23.574
$f_{10}$	Dn.	21.052	20.258	18.544	15.064	12.444	9.306	6.129	3.000	2.062
	Rcv.	8.948	9.742	11.456	14.936	17.556	20.694	23.871	27.000	27.938
$f_{11}$	Dn.	21.812	21.176	20.082	18.496	16.350	14.174	11.896	8.586	6.818
	Rcv.	8.188	8.824	9.918	11.504	13.650	15.826	18.104	21.414	23.182
$f_{12}$	Dn.	21.000	20.000	18.000	15.000	12.000	9.000	6.000	3.000	2.000
	Rcv.	9.000	10.000	12.000	15.000	18.000	21.000	24.000	27.000	28.000

**Table 4:** Average execution times of pIPA and IPA for 100-dimensional problems.

Fn.		pIPA									IPA	
		** prc **									** NoD **	** NoR **
		**30**	**35**	**40**	**50**	**60**	**70**	**80**	**90**	**95**	**1**	**1**
$f_{1}$	Mean	0.369	0.331	0.295	0.317	0.292	0.245	0.229	0.186	0.154	0.103	
	Std.	$1.528\times 10^{-1}$	$1.688\times 10^{-1}$	$8.445\times 10^{-2}$	$9.433\times 10^{-2}$	$1.145\times 10^{-1}$	$8.012\times 10^{-2}$	$1.000\times 10^{-1}$	$8.897\times 10^{-2}$	$6.730\times 10^{-2}$	$8.757\times 10^{-3}$	
$f_{2}$	Mean	0.302	0.338	0.310	0.338	0.292	0.327	0.239	0.171	0.149	0.124	
	Std.	$8.364\times 10^{-2}$	$1.419\times 10^{-1}$	$9.550\times 10^{-2}$	$1.312\times 10^{-1}$	$1.113\times 10^{-1}$	$1.944\times 10^{-1}$	$1.090\times 10^{-1}$	$5.980\times 10^{-2}$	$7.212\times 10^{-2}$	$1.382\times 10^{-2}$	
$f_{3}$	Mean	1.864	1.851	1.829	1.887	1.897	1.762	1.712	1.692	1.673	1.550	
	Std.	$1.461\times 10^{-1}$	$1.382\times 10^{-1}$	$1.033\times 10^{-1}$	$2.180\times 10^{-1}$	$1.832\times 10^{-1}$	$1.000\times 10^{-1}$	$1.172\times 10^{-1}$	9.750 × 10^−2^	1.078 × 10^−1^	3.023 × 10^−2^	
$f_{4}$	Mean	0.261	0.265	0.252	0.272	0.249	0.209	0.190	0.162	0.121	0.102	
	Std.	$1.043\times 10^{-1}$	$8.713\times 10^{-2}$	$9.100\times 10^{-2}$	$9.272\times 10^{-2}$	$8.086\times 10^{-2}$	$6.104\times 10^{-2}$	$9.397\times 10^{-2}$	$9.970\times 10^{-2}$	$5.727\times 10^{-2}$	$9.404\times 10^{-3}$	
$f_{5}$	Mean	0.364	0.361	0.358	0.370	0.328	0.306	0.268	0.241	0.212	0.202	
	Std.	$1.070\times 10^{-1}$	$9.662\times 10^{-2}$	$8.159\times 10^{-2}$	$1.306\times 10^{-1}$	$9.625\times 10^{-2}$	$7.606\times 10^{-2}$	$6.572\times 10^{-2}$	$7.230\times 10^{-2}$	$6.779\times 10^{-2}$	$2.201\times 10^{-2}$	
$f_{6}$	Mean	0.814	0.866	0.815	0.881	0.849	0.834	0.839	0.787	0.771	0.721	
	Std.	$1.280\times 10^{-1}$	$1.978\times 10^{-1}$	$1.335\times 10^{-1}$	$2.230\times 10^{-1}$	$1.999\times 10^{-1}$	$1.613\times 10^{-1}$	$1.657\times 10^{-1}$	$1.494\times 10^{-1}$	$1.570\times 10^{-1}$	$2.621\times 10^{-2}$	
$f_{7}$	Mean	0.755	0.752	0.748	0.746	0.734	0.723	0.649	0.615	0.611	0.620	
	Std.	$1.760\times 10^{-1}$	$1.373\times 10^{-1}$	$1.590\times 10^{-1}$	$1.210\times 10^{-1}$	$1.362\times 10^{-1}$	$1.607\times 10^{-1}$	$1.005\times 10^{-1}$	$1.174\times 10^{-1}$	$1.172\times 10^{-1}$	$2.226\times 10^{-2}$	
$f_{8}$	Mean	0.790	0.819	0.765	0.849	0.771	0.746	0.720	0.685	0.669	0.665	
	Std.	$1.449\times 10^{-1}$	$1.735\times 10^{-1}$	$1.327\times 10^{-1}$	$1.822\times 10^{-1}$	$1.489\times 10^{-1}$	$1.565\times 10^{-1}$	$1.247\times 10^{-1}$	$1.282\times 10^{-1}$	$1.645\times 10^{-1}$	$2.196\times 10^{-2}$	
$f_{9}$	Mean	0.709	0.750	0.730	0.774	0.720	0.699	0.683	0.600	0.650	0.600	
	Std.	$1.324\times 10^{-1}$	$1.870\times 10^{-1}$	$1.685\times 10^{-1}$	$2.078\times 10^{-1}$	$1.620\times 10^{-1}$	$1.549\times 10^{-1}$	$1.338\times 10^{-1}$	$1.109\times 10^{-1}$	$1.423\times 10^{-1}$	$4.716\times 10^{-2}$	
$f_{10}$	Mean	1.199	1.060	1.124	1.196	1.104	1.118	1.177	0.949	1.017	1.003	
	Std.	$2.481\times 10^{-1}$	$1.460\times 10^{-1}$	$2.100\times 10^{-1}$	$2.723\times 10^{-1}$	$2.000\times 10^{-1}$	$2.774\times 10^{-1}$	$2.293\times 10^{-1}$	$1.168\times 10^{-1}$	$1.913\times 10^{-1}$	$1.192\times 10^{-2}$	
$f_{11}$	Mean	1.446	1.451	1.448	1.425	1.419	1.381	1.387	1.374	1.375	1.239	
	Std.	$1.350\times 10^{-1}$	$1.449\times 10^{-1}$	$1.467\times 10^{-1}$	$1.375\times 10^{-1}$	$1.301\times 10^{-1}$	$1.155\times 10^{-1}$	$1.259\times 10^{-1}$	$1.395\times 10^{-1}$	$1.598\times 10^{-1}$	$2.487\times 10^{-2}$	
$f_{12}$	Mean	1.802	1.785	1.767	1.752	1.724	1.707	1.629	1.645	1.628	1.513	
	Std.	$1.223\times 10^{-1}$	$1.145\times 10^{-1}$	$1.172\times 10^{-1}$	$1.386\times 10^{-1}$	$1.595\times 10^{-1}$	$1.683\times 10^{-1}$	$9.727\times 10^{-2}$	$1.817\times 10^{-1}$	$6.466\times 10^{-2}$	$3.417\times 10^{-2}$	

**Table 5:** Comparison between pIPA and other meta-heuristics for 100-dimensional problems.

Fn.		pIPA	IPA	MFO	PSO	GSA	BA	FPA	SMS	FA	GA
$f_{1}$	Mean	$4.6656\times 10^{-49}$	$7.4671\times 10^{-27}$	$1.1700\times 10^{-4}$	$1.3212\times 10^{0}$	$6.0823\times 10^{2}$	$2.0792\times 10^{4}$	$2.0364\times 10^{2}$	$1.2000\times 10^{2}$	$7.4807\times 10^{3}$	$2.1886\times 10^{4}$
	Std.	$8.7556\times 10^{-49}$	$1.9330\times 10^{-26}$	$1.5000\times 10^{-4}$	$1.1539\times 10^{0}$	$4.6465\times 10^{2}$	$5.8924\times 10^{3}$	$7.8398\times 10^{1}$	$0.0000\times 10^{0}$	$8.9485\times 10^{2}$	$2.8796\times 10^{3}$
	Rank	1	2	3	4	7	9	6	5	8	10
$f_{2}$	Mean	$2.0915\times 10^{-35}$	$9.2362\times 10^{-26}$	$6.3900\times 10^{-4}$	$7.7156\times 10^{0}$	$2.2753\times 10^{1}$	$8.9786\times 10^{1}$	$1.1169\times 10^{1}$	$2.0531\times 10^{-2}$	$3.9325\times 10^{1}$	$5.6518\times 10^{1}$
	Std.	$3.9040\times 10^{-35}$	$2.2260\times 10^{-25}$	$8.7700\times 10^{-4}$	$4.1321\times 10^{0}$	$3.3651\times 10^{0}$	$4.1958\times 10^{1}$	$2.9196\times 10^{0}$	$4.7180\times 10^{-3}$	$2.4659\times 10^{0}$	$5.6609\times 10^{0}$
	Rank	1	2	3	4	7	10	6	4	8	9
$f_{3}$	Mean	$9.3947\times 10^{-30}$	$4.5045\times 10^{-14}$	$6.9673\times 10^{2}$	$7.3639\times 10^{2}$	$1.3576\times 10^{5}$	$6.2481\times 10^{4}$	$2.3757\times 10^{2}$	$3.7820\times 10^{4}$	$1.7357\times 10^{4}$	$3.7010\times 10^{4}$
	Std.	$3.3014\times 10^{-29}$	$2.4546\times 10^{-13}$	$1.8853\times 10^{2}$	$3.6178\times 10^{2}$	$4.8653\times 10^{4}$	$2.9769\times 10^{4}$	$1.3665\times 10^{2}$	$0.0000\times 10^{0}$	$1.7401\times 10^{3}$	$5.5722\times 10^{3}$
	Rank	1	2	4	5	10	9	3	8	6	7
$f_{4}$	Mean	$3.8292\times 10^{0}$	$2.3461\times 10^{1}$	$7.0686\times 10^{1}$	$1.2973\times 10^{1}$	$7.8782\times 10^{1}$	$4.9743\times 10^{1}$	$1.2573\times 10^{1}$	$6.9170\times 10^{1}$	$3.3954\times 10^{1}$	$5.9143\times 10^{1}$
	Std.	$4.2740\times 10^{0}$	$1.6448\times 10^{1}$	$5.2751\times 10^{0}$	$2.6344\times 10^{0}$	$2.8141\times 10^{0}$	$1.0144\times 10^{1}$	$2.2900\times 10^{0}$	$3.8767\times 10^{0}$	$1.8697\times 10^{0}$	$4.6485\times 10^{0}$
	Rank	1	4	9	3	10	6	2	8	5	7
$f_{5}$	Mean	$9.8531\times 10^{1}$	$1.1020\times 10^{2}$	$1.3915\times 10^{2}$	$7.7361\times 10^{4}$	$7.4100\times 10^{2}$	$1.9951\times 10^{6}$	$1.0975\times 10^{4}$	$6.3822\times 10^{6}$	$3.7950\times 10^{6}$	$3.1321\times 10^{7}$
	Std.	$4.4030\times 10^{-1}$	$4.3071\times 10^{1}$	$1.2026\times 10^{2}$	$5.1156\times 10^{4}$	$7.8124\times 10^{2}$	$1.2524\times 10^{6}$	$1.2057\times 10^{4}$	$7.2997\times 10^{5}$	$7.5903\times 10^{5}$	$5.2645\times 10^{6}$
	Rank	1	2	3	6	4	7	5	9	8	10
$f_{6}$	Mean	$0.0000\times 10^{0}$	$0.0000\times 10^{0}$	$1.1300\times 10^{-4}$	$2.8665\times 10^{2}$	$3.0810\times 10^{3}$	$1.7053\times 10^{4}$	$1.7538\times 10^{2}$	$4.1439\times 10^{4}$	$7.8287\times 10^{3}$	$2.0965\times 10^{4}$
	Std.	$0.0000\times 10^{0}$	$0.0000\times 10^{0}$	$9.8700\times 10^{-5}$	$1.0708\times 10^{2}$	$8.9863\times 10^{2}$	$4.9176\times 10^{3}$	$6.3453\times 10^{1}$	$3.2952\times 10^{3}$	$9.7521\times 10^{2}$	$3.8681\times 10^{3}$
	Rank	1	1	3	5	6	8	4	10	7	9
$f_{7}$	Mean	$1.1218\times 10^{-4}$	$7.0755\times 10^{-3}$	$9.1155\times 10^{-2}$	$1.0373\times 10^{0}$	$1.1298\times 10^{-1}$	$6.0451\times 10^{0}$	$1.3594\times 10^{-1}$	$4.9520\times 10^{-2}$	$1.9063\times 10^{0}$	$1.3375\times 10^{1}$
	Std.	$1.2010\times 10^{-4}$	$9.4016\times 10^{-3}$	$4.6420\times 10^{-2}$	$3.1032\times 10^{-1}$	$3.7607\times 10^{-2}$	$3.0453\times 10^{0}$	$6.1212\times 10^{-2}$	$2.4015\times 10^{-2}$	$4.6006\times 10^{-1}$	$3.0815\times 10^{0}$
	Rank	1	2	4	7	5	9	6	3	8	10
$f_{8}$	Mean	$-2.3315\times 10^{4}$	$-2.5129\times 10^{4}$	$-8.4968\times 10^{3}$	$-3.5710\times 10^{3}$	$-2.3523\times 10^{3}$	$6.5535\times 10^{4}$	$-8.0867\times 10^{3}$	$-3.9428\times 10^{3}$	$-3.6621\times 10^{3}$	$-6.3312\times 10^{3}$
	Std.	$5.5671\times 10^{2}$	$9.5538\times 10^{2}$	$7.2587\times 10^{2}$	$4.3080\times 10^{2}$	$3.8217\times 10^{2}$	$0.0000\times 10^{0}$	$1.5535\times 10^{2}$	$4.0416\times 10^{2}$	$2.1416\times 10^{2}$	$3.3257\times 10^{2}$
	Rank	2	1	3	8	9	10	4	6	7	5
$f_{9}$	Mean	$0.0000\times 10^{0}$	$0.0000\times 10^{0}$	$8.4600\times 10^{1}$	$1.2430\times 10^{2}$	$3.1000\times 10^{1}$	$9.6215\times 10^{1}$	$9.2692\times 10^{1}$	$1.5284\times 10^{2}$	$2.1490\times 10^{2}$	$2.3683\times 10^{2}$
	Std.	$0.0000\times 10^{0}$	$0.0000\times 10^{0}$	$1.6167\times 10^{1}$	$1.4251\times 10^{1}$	$1.3661\times 10^{1}$	$1.9588\times 10^{1}$	$1.4224\times 10^{1}$	$1.8554\times 10^{1}$	$1.7219\times 10^{1}$	$1.9034\times 10^{1}$
	Rank	1	1	4	7	3	6	5	8	9	10
$f_{10}$	Mean	$1.2730\times 10^{-15}$	$6.9604\times 10^{-14}$	$1.2604\times 10^{0}$	$9.1679\times 10^{0}$	$3.7410\times 10^{0}$	$1.5946\times 10^{1}$	$6.8448\times 10^{0}$	$1.9133\times 10^{1}$	$1.4568\times 10^{1}$	$1.7846\times 10^{1}$
	Std.	$2.7492\times 10^{-15}$	$1.7583\times 10^{-13}$	$7.2956\times 10^{-1}$	$1.5690\times 10^{0}$	$1.7127\times 10^{-1}$	$7.7495\times 10^{-1}$	$1.2500\times 10^{0}$	$2.3852\times 10^{-1}$	$4.6751\times 10^{-1}$	$5.3115\times 10^{-1}$
	Rank	1	2	3	6	4	8	5	10	7	9
$f_{11}$	Mean	$0.0000\times 10^{0}$	$0.0000\times 10^{0}$	$1.9080\times 10^{-2}$	$1.2419\times 10^{1}$	$4.8683\times 10^{-1}$	$2.2028\times 10^{2}$	$2.7161\times 10^{0}$	$4.2053\times 10^{2}$	$6.9658\times 10^{1}$	$1.7990\times 10^{2}$
	Std.	$0.0000\times 10^{0}$	$0.0000\times 10^{0}$	$2.1732\times 10^{-2}$	$4.1658\times 10^{0}$	$4.9785\times 10^{-2}$	$5.4707\times 10^{1}$	$7.2772\times 10^{-1}$	$2.5256\times 10^{1}$	$1.2114\times 10^{1}$	$3.2440\times 10^{1}$
	Rank	1	1	3	6	4	9	5	10	7	8
$f_{12}$	Mean	$7.2542\times 10^{-1}$	$8.1573\times 10^{3}$	$8.9401\times 10^{-1}$	$1.3874\times 10^{1}$	$4.6344\times 10^{-1}$	$2.8934\times 10^{7}$	$4.1053\times 10^{0}$	$8.7428\times 10^{6}$	$3.6840\times 10^{5}$	$3.4132\times 10^{7}$
	Std.	$7.2941\times 10^{-2}$	$4.4671\times 10^{4}$	$8.8127\times 10^{-1}$	$5.8537\times 10^{0}$	$1.3760\times 10^{-1}$	$2.1787\times 10^{6}$	$1.0435\times 10^{0}$	$1.4057\times 10^{6}$	$1.7213\times 10^{5}$	$1.8934\times 10^{6}$
	Rank	2	6	3	5	1	9	4	8	7	10
Average		1.1667	2.1667	3.7500	5.5000	5.8333	8.3333	4.5833	7.4167	7.2500	8.6667
Overall		1	2	3	5	6	9	4	8	7	10

**Table 6:** Sr and Me metrics of pIPA and IPA for 100-dimensional problems.

Fn.		pIPA									IPA	
		prc									NoD	NoR
		**30**	**35**	**40**	**50**	**60**	**70**	**80**	**90**	**95**	**1**	**1**
$f_{1}$	Sr	96.667	100.000	100.000	100.000	100.000	100.000	100.000	100.000	100.000	100.000	
	Me	28,932.621	27,543.900	25,436.567	23,799.533	22,793.033	22,244.667	21,703.133	22,680.300	23,700.467	27,523.200	
$f_{2}$	Sr	0.000	0.000	0.000	90.000	100.000	100.000	100.000	100.000	100.000	76.667	
	Me	-	-	-	29,333.630	27,982.800	26,358.233	25,839.000	25,447.733	26,076.167	28,915.478	
$f_{3}$	Sr	56.667	83.333	100.000	0.000	0.000	0.000	0.000	0.000	0.000	0.000	
	Me	29,221.000	28,886.640	27484.367	-	-	-	-	-	-	-	
$f_{4}$	Sr	93.333	93.333	83.333	0.000	0.000	0.000	0.000	0.000	0.000	0.000	
	Me	28,668.393	29,285.179	28,725.320	-	-	-	-	-	-	-	
$f_{5}$	Sr	100.000	100.000	100.000	100.000	100.000	100.000	100.000	100.000	100.000	93.333	
	Me	20,766.733	19,076.967	15,832.333	13,069.933	12,149.933	12,800.167	12,469.800	13,561.267	16,181.633	23,812.929	
$f_{6}$	Sr	100.000	100.000	100.000	100.000	100.000	100.000	100.000	100.000	100.000	100.000	
	Me	20,649.900	18,434.000	15,241.500	10,976.333	10,661.867	10,339.733	10,223.667	10,728.667	11,853.900	15,042.667	
$f_{7}$	Sr	100.000	100.000	100.000	36.667	53.333	63.333	56.667	53.333	56.667	13.333	
	Me	22,497.400	21,957.967	17,780.633	22,587.455	21,852.875	23,439.579	22,875.235	26,454.125	27,074.529	23,552.500	
$f_{8}$	Sr	100.000	100.000	100.000	100.000	100.000	100.000	100.000	100.000	100.000	100.000	
	Me	45.933	47.433	48.133	52.933	50.233	44.667	46.433	50.433	66.967	83.667	
$f_{9}$	Sr	100.000	100.000	100.000	100.000	100.000	100.000	100.000	100.000	83.333	16.667	
	Me	24,964.933	23,534.733	20,910.733	17,939.767	17,425.800	16,729.700	16,520.900	17,523.367	19,559.440	23,936.000	
$f_{10}$	Sr	100.000	100.000	100.000	3.333	16.667	10.000	3.333	0.000	3.333	100.000	
	Me	27,385.900	26,141.567	25,172.067	21,276.000	20,665.600	20,560.000	19,533.000	-	22,629.000	25,711.100	
$f_{11}$	Sr	100.000	100.000	100.000	100.000	100.000	100.000	100.000	100.000	100.000	100.000	
	Me	26,010.900	24,848.133	22,339.100	19,861.567	19,397.500	18,708.400	18,104.267	19,059.567	20,486.933	23,728.100	
$f_{12}$	Sr	0.000	0.000	3.333	100.000	100.000	100.000	100.000	100.000	96.667	30.000	
	Me	-	-	14,521.000	14,527.300	15,791.333	15,502.900	15,845.100	17,779.567	20,178.552	26,866.000	

**Table 7:** Statistical comparison between pIPA and other algorithms for 100-dimensional problems.

**pIPA vs.**	**Z-val.**	R+	R−	ρ **-val.**	**Sign.**
IPA	−2.1917	4	11	-	-
MFO	−3.0594	0	78	0.0022	pIPA
PSO	−3.0594	0	78	0.0022	pIPA
GSA	−2.9025	2	76	0.0037	pIPA
BA	−3.0594	0	78	0.0022	pIPA
FPA	−3.0594	0	78	0.0022	pIPA
SMS	−3.0594	0	78	0.0022	pIPA
FA	−3.0594	0	78	0.0022	pIPA
GA	−3.0594	0	78	0.0022	pIPA

**Table 8:** Results of pIPA with different prc values for 200-dimensional problems.

Fn.		prc								
		**30**	**35**	**40**	**50**	**60**	**70**	**80**	**90**	**95**
$f_{1}$	Mean	$1.1215\times 10^{-182}$	$2.8211\times 10^{-219}$	$1.7872\times 10^{-246}$	$2.8169\times 10^{-270}$	$1.5992\times 10^{-294}$	$0.0000\times 10^{0}$	$0.0000\times 10^{0}$	$0.0000\times 10^{0}$	$0.0000\times 10^{0}$
	Std.	$0.0000\times 10^{0}$	$0.0000\times 10^{0}$	$0.0000\times 10^{0}$	$0.0000\times 10^{0}$	$0.0000\times 10^{0}$	$0.0000\times 10^{0}$	$0.0000\times 10^{0}$	$0.0000\times 10^{0}$	$0.0000\times 10^{0}$
$f_{2}$	Mean	$2.8799\times 10^{-99}$	$1.1070\times 10^{-117}$	$2.4705\times 10^{-131}$	$2.0965\times 10^{-156}$	$2.4892\times 10^{-172}$	$1.1690\times 10^{-190}$	$3.3511\times 10^{-211}$	$3.0729\times 10^{-231}$	$8.6019\times 10^{-230}$
	Std.	$7.2337\times 10^{-99}$	$2.6396\times 10^{-117}$	$9.5837\times 10^{-131}$	$6.4228\times 10^{-156}$	$0.0000\times 10^{0}$	$0.0000\times 10^{0}$	$0.0000\times 10^{0}$	$0.0000\times 10^{0}$	$0.0000\times 10^{0}$
$f_{3}$	Mean	$3.7471\times 10^{-178}$	$4.6140\times 10^{-208}$	$1.5437\times 10^{-230}$	$1.1330\times 10^{-209}$	$2.1382\times 10^{-198}$	$3.0109\times 10^{-215}$	$1.6846\times 10^{-166}$	$1.8593\times 10^{3}$	$4.0131\times 10^{4}$
	Std.	$0.0000\times 10^{0}$	$0.0000\times 10^{0}$	$0.0000\times 10^{0}$	$0.0000\times 10^{0}$	$0.0000\times 10^{0}$	$0.0000\times 10^{0}$	$0.0000\times 10^{0}$	$9.1681\times 10^{3}$	$5.6737\times 10^{4}$
$f_{4}$	Mean	$1.4745\times 10^{-3}$	$8.5540\times 10^{-4}$	$6.7365\times 10^{-5}$	$6.7504\times 10^{1}$	$9.2555\times 10^{1}$	$9.2763\times 10^{1}$	$9.2663\times 10^{1}$	$9.2800\times 10^{1}$	$9.2963\times 10^{1}$
	Std.	$2.0434\times 10^{-3}$	$1.8510\times 10^{-3}$	$7.4571\times 10^{-5}$	$4.1415\times 10^{1}$	$1.1149\times 10^{0}$	$9.8683\times 10^{-1}$	$1.1799\times 10^{0}$	$1.1458\times 10^{0}$	$1.7564\times 10^{0}$
$f_{5}$	Mean	$1.9882\times 10^{2}$	$1.9882\times 10^{2}$	$1.9876\times 10^{2}$	$1.9759\times 10^{2}$	$1.9775\times 10^{2}$	$1.9754\times 10^{2}$	$1.9785\times 10^{2}$	$1.9767\times 10^{2}$	$1.9786\times 10^{2}$
	Std.	$9.2636\times 10^{-2}$	$1.6440\times 10^{-1}$	$1.2063\times 10^{-1}$	$7.4994\times 10^{-1}$	$6.2291\times 10^{-1}$	$7.5397\times 10^{-1}$	$5.3892\times 10^{-1}$	$5.9048\times 10^{-1}$	$5.7341\times 10^{-1}$
$f_{6}$	Mean	$0.0000\times 10^{0}$	$0.0000\times 10^{0}$	$0.0000\times 10^{0}$	$0.0000\times 10^{0}$	$0.0000\times 10^{0}$	$0.0000\times 10^{0}$	$0.0000\times 10^{0}$	$0.0000\times 10^{0}$	$0.0000\times 10^{0}$
	Std.	$0.0000\times 10^{0}$	$0.0000\times 10^{0}$	$0.0000\times 10^{0}$	$0.0000\times 10^{0}$	$0.0000\times 10^{0}$	$0.0000\times 10^{0}$	$0.0000\times 10^{0}$	$0.0000\times 10^{0}$	$0.0000\times 10^{0}$
$f_{7}$	Mean	$5.8805\times 10^{-11}$	$1.6428\times 10^{-9}$	$1.2257\times 10^{-10}$	$5.2013\times 10^{-5}$	$6.2282\times 10^{-5}$	$4.6898\times 10^{-5}$	$4.5043\times 10^{-5}$	$3.4159\times 10^{-5}$	$5.6158\times 10^{-5}$
	Std.	$2.2231\times 10^{-10}$	$7.1936\times 10^{-9}$	$3.7133\times 10^{-10}$	$5.1521\times 10^{-5}$	$6.7997\times 10^{-5}$	$5.6567\times 10^{-5}$	$3.7871\times 10^{-5}$	$4.4515\times 10^{-5}$	$5.5912\times 10^{-5}$
$f_{8}$	Mean	$-2.7710\times 10^{4}$	$-2.7707\times 10^{4}$	$-2.7642\times 10^{4}$	$-5.2524\times 10^{4}$	$-5.3241\times 10^{4}$	$-5.4380\times 10^{4}$	$-5.5547\times 10^{4}$	$-5.6822\times 10^{4}$	$-5.7392\times 10^{4}$
	Std.	$5.9042\times 10^{2}$	$5.5372\times 10^{2}$	$5.1354\times 10^{2}$	$5.0406\times 10^{2}$	$5.4389\times 10^{2}$	$6.0775\times 10^{2}$	$4.8143\times 10^{2}$	$7.2056\times 10^{2}$	$6.4177\times 10^{2}$
$f_{9}$	Mean	$0.0000\times 10^{0}$	$0.0000\times 10^{0}$	$0.0000\times 10^{0}$	$0.0000\times 10^{0}$	$0.0000\times 10^{0}$	$0.0000\times 10^{0}$	$0.0000\times 10^{0}$	$0.0000\times 10^{0}$	$8.1740\times 10^{0}$
	Std.	$0.0000\times 10^{0}$	$0.0000\times 10^{0}$	$0.0000\times 10^{0}$	$0.0000\times 10^{0}$	$0.0000\times 10^{0}$	$0.0000\times 10^{0}$	$0.0000\times 10^{0}$	$0.0000\times 10^{0}$	$4.4770\times 10^{1}$
$f_{10}$	Mean	$4.4408\times 10^{-16}$	$4.4408\times 10^{-16}$	$4.4408\times 10^{-16}$	$1.9850\times 10^{1}$	$1.9820\times 10^{1}$	$1.9760\times 10^{1}$	$1.9702\times 10^{1}$	$1.8912\times 10^{1}$	$1.8881\times 10^{1}$
	Std.	$1.0029\times 10^{-31}$	$1.0029\times 10^{-31}$	$1.0029\times 10^{-31}$	$2.0332\times 10^{-2}$	$1.8486\times 10^{-2}$	$3.8931\times 10^{-2}$	$4.7974\times 10^{-2}$	$3.5734\times 10^{0}$	$3.5692\times 10^{0}$
$f_{11}$	Mean	$0.0000\times 10^{0}$	$0.0000\times 10^{0}$	$0.0000\times 10^{0}$	$0.0000\times 10^{0}$	$0.0000\times 10^{0}$	$0.0000\times 10^{0}$	$0.0000\times 10^{0}$	$0.0000\times 10^{0}$	$0.0000\times 10^{0}$
	Std.	$0.0000\times 10^{0}$	$0.0000\times 10^{0}$	$0.0000\times 10^{0}$	$0.0000\times 10^{0}$	$0.0000\times 10^{0}$	$0.0000\times 10^{0}$	$0.0000\times 10^{0}$	$0.0000\times 10^{0}$	$0.0000\times 10^{0}$
$f_{12}$	Mean	$1.1439\times 10^{0}$	$1.1261\times 10^{0}$	$1.0911\times 10^{0}$	$2.4680\times 10^{-1}$	$1.9084\times 10^{-1}$	$1.4524\times 10^{-1}$	$1.2154\times 10^{-1}$	$1.1938\times 10^{-1}$	$1.9465\times 10^{-1}$
	Std.	$1.6359\times 10^{-2}$	$1.2593\times 10^{-2}$	$6.9926\times 10^{-3}$	$2.4716\times 10^{-2}$	$2.7305\times 10^{-2}$	$1.7195\times 10^{-2}$	$1.9028\times 10^{-2}$	$1.9587\times 10^{-2}$	$5.0395\times 10^{-2}$

**Table 9:** Average number of donors and receivers for 200-dimensional problems.

Fn.		prc								
		**30**	**35**	**40**	**50**	**60**	**70**	**80**	**90**	**95**
$f_{1}$	Dn.	70.000	65.000	60.000	50.000	40.000	31.343	24.311	15.852	9.420
	Rcv.	30.000	35.000	40.000	50.000	60.000	68.657	75.689	84.148	90.580
$f_{2}$	Dn.	70.000	65.000	60.000	50.000	40.000	30.000	20.000	10.000	5.000
	Rcv.	30.000	35.000	40.000	50.000	60.000	70.000	80.000	90.000	95.000
$f_{3}$	Dn.	70.000	65.000	60.000	50.000	40.000	30.000	20.000	10.000	5.000
	Rcv.	30.000	35.000	40.000	50.000	60.000	70.000	80.000	90.000	95.000
$f_{4}$	Dn.	99.935	99.835	99.732	63.163	40.011	30.019	20.012	10.002	5.000
	Rcv.	0.065	0.165	0.268	36.837	59.989	69.981	79.988	89.998	95.000
$f_{5}$	Dn.	70.000	65.000	60.000	50.000	40.000	30.000	20.000	10.000	5.000
	Rcv.	30.000	35.000	40.000	50.000	60.000	70.000	80.000	90.000	95.000
$f_{6}$	Dn.	75.762	73.184	71.157	68.889	66.870	64.581	61.846	58.152	55.175
	Rcv.	24.238	26.816	28.843	31.111	33.130	35.419	38.154	41.848	44.825
$f_{7}$	Dn.	70.000	65.000	60.000	50.000	40.000	30.000	20.000	10.000	5.000
	Rcv.	30.000	35.000	40.000	50.000	60.000	70.000	80.000	90.000	95.000
$f_{8}$	Dn.	70.000	65.000	60.000	50.000	40.000	30.000	20.000	10.000	5.000
	Rcv.	30.000	35.000	40.000	50.000	60.000	70.000	80.000	90.000	95.000
$f_{9}$	Dn.	75.626	73.034	70.960	68.453	65.456	62.104	58.387	53.313	47.864
	Rcv.	24.374	26.966	29.040	31.547	34.544	37.896	41.613	46.687	52.136
$f_{10}$	Dn.	75.344	72.676	70.188	50.000	40.000	30.000	20.000	11.278	5.001
	Rcv.	24.656	27.324	29.812	50.000	60.000	70.000	80.000	88.722	94.999
$f_{11}$	Dn.	75.696	73.098	71.006	65.040	65.088	61.427	57.289	52.021	47.660
	Rcv.	24.304	26.902	28.994	34.960	34.912	38.573	42.711	47.979	52.340
$f_{12}$	Dn.	70.000	65.000	60.000	50.000	40.000	30.000	20.000	10.000	5.000
	Rcv.	30.000	35.000	40.000	50.000	60.000	70.000	80.000	90.000	95.000

**Table 10:** Comparison between pIPA and other meta-heuristics for 200-dimensional problems.

Fn.		pIPA	IPA	ALO	PSO	SMS	BA	FPA	CS	FA	GA
$f_{1}$	Mean	$0.0000\times 10^{0}$	$1.1523\times 10^{-189}$	$7.8900\times 10^{-07}$	$2.3799\times 10^{1}$	$1.0392\times 10^{3}$	$1.1173\times 10^{3}$	$5.5989\times 10^{1}$	$3.8000\times 10^{-05}$	$7.6128\times 10^{1}$	$2.2775\times 10^{2}$
	Std.	$0.0000\times 10^{0}$	$0.0000\times 10^{0}$	$1.1000\times 10^{-07}$	$1.1721\times 10^{1}$	$4.2430\times 10^{-1}$	$2.0731\times 10^{4}$	$3.2678\times 10^{1}$	$1.8500\times 10^{-5}$	$1.57444\times 10^{0}$	$1.8656\times 10^{2}$
	Rank	1	2	3	5	9	10	6	4	7	8
$f_{2}$	Mean	$3.0729\times 10^{-231}$	$2.0701\times 10^{-144}$	$5.3082\times 10^{2}$	$2.3787\times 10^{2}$	$1.8324\times 10^{3}$	$3.8428\times 10^{3}$	$2.8060\times 10^{2}$	$4.0010\times 10^{2}$	$6.1119\times 10^{2}$	$6.3226\times 10^{3}$
	Std.	$0.0000\times 10^{0}$	$1.1305\times 10^{-143}$	$2.2267\times 10^{2}$	$2.2432\times 10^{1}$	$1.2200\times 10^{-2}$	$4.6828\times 10^{2}$	$6.9384\times 10^{0}$	$8.6560\times 10^{-1}$	$7.1219\times 10^{1}$	$1.0927\times 10^{3}$
	Rank	1	2	6	3	8	9	4	5	7	10
$f_{3}$	Mean	$1.5437\times 10^{-230}$	$1.6826\times 10^{5}$	$2.3314\times 10^{3}$	$4.6933\times 10^{3}$	$2.0349\times 10^{3}$	$1.0908\times 10^{3}$	$2.4219\times 10^{4}$	$1.2957\times 10^{4}$	$1.4852\times 10^{4}$	$1.1206\times 10^{4}$
	Std.	$0.0000\times 10^{0}$	$1.3627\times 10^{5}$	$5.0718\times 10^{2}$	$5.0357\times 10^{2}$	$3.7800\times 10^{-1}$	$4.7506\times 10^{2}$	$8.5400\times 10^{3}$	$6.3375\times 10^{2}$	$6.4184\times 10^{3}$	$3.9861\times 10^{3}$
	Rank	1	10	4	5	3	2	9	7	8	6
$f_{4}$	Mean	$6.7365\times 10^{-5}$	$8.3428\times 10^{1}$	$3.0580\times 10^{1}$	$4.0111\times 10^{1}$	$3.0026\times 10^{2}$	$6.5667\times 10^{1}$	$3.7689\times 10^{1}$	$3.0936\times 10^{1}$	$2.7360\times 10^{0}$	$1.0154\times 10^{2}$
	Std.	$7.4571\times 10^{-5}$	$1.2702\times 10^{0}$	$1.1446\times 10^{0}$	$5.8790\times 10^{-1}$	$2.3000\times 10^{-3}$	$2.8293\times 10^{0}$	$2.4572\times 10^{0}$	$1.6877\times 10^{0}$	$5.4729\times 10^{-1}$	$2.5321\times 10^{0}$
	Rank	1	9	4	7	3	8	6	5	2	10
$f_{5}$	Mean	$1.9767\times 10^{2}$	$1.9816\times 10^{2}$	$1.6704\times 10^{2}$	$9.1123\times 10^{2}$	$3.8635\times 10^{3}$	$1.4108\times 10^{3}$	$3.1507\times 10^{3}$	$3.3267\times 10^{2}$	$1.3217\times 10^{3}$	$9.6449\times 10^{2}$
	Std.	$5.9048\times 10^{-1}$	$3.9420\times 10^{-1}$	$4.9746\times 10^{1}$	$9.5245\times 10^{1}$	$5.3290\times 10^{-1}$	$5.9107\times 10^{2}$	$1.4906\times 10^{3}$	$1.5988\times 10^{2}$	$1.1476\times 10^{2}$	$7.4876\times 10^{2}$
	Rank	2	3	1	5	10	8	9	4	7	6
$f_{6}$	Mean	$0.0000\times 10^{0}$	$0.0000\times 10^{0}$	$7.6000\times 10^{-7}$	$4.3421\times 10^{1}$	$2.4944\times 10^{3}$	$5.1206\times 10^{1}$	$1.6699\times 10^{2}$	$8.1700\times 10^{-5}$	$7.8420\times 10^{1}$	$4.8256\times 10^{2}$
	Std.	$0.0000\times 10^{0}$	$0.0000\times 10^{0}$	$7.3900\times 10^{-8}$	$1.4206\times 10^{1}$	$3.0000\times 10^{-4}$	$1.2005\times 10^{1}$	$4.1109\times 10^{1}$	$4.5500\times 10^{-5}$	$2.3405\times 10^{0}$	$2.7861\times 10^{2}$
	Rank	1	1	3	5	10	6	8	4	7	9
$f_{7}$	Mean	$1.2257\times 10^{-10}$	$7.7848\times 10^{-4}$	$5.0546\times 10^{-2}$	$1.7321\times 10^{1}$	$2.8359\times 10^{1}$	$2.4344\times 10^{0}$	$4.8391\times 10^{0}$	$4.0131\times 10^{-1}$	$2.7300\times 10^{-2}$	$1.1656\times 10^{2}$
	Std.	$3.7133\times 10^{-10}$	$1.1574\times 10^{-3}$	$1.4407\times 10^{-2}$	$4.0133\times 10^{0}$	$1.9900\times 10^{-5}$	$1.2756\times 10^{-1}$	$1.5354\times 10^{0}$	$8.7070\times 10^{-3}$	$4.1100\times 10^{-3}$	$6.016\times 10^{1}$
	Rank	1	2	4	8	9	6	7	5	3	10
$f_{8}$	Mean	$-5.6822\times 10^{4}$	$-6.1749\times 10^{4}$	$-4.4426\times 10^{4}$	$-1.8136\times 10^{4}$	$-3.5969\times 10^{4}$	$-2.5632\times 10^{4}$	$-4.5771\times 10^{4}$	$-5.2600\times 10^{4}$	$-3.9753\times 10^{4}$	$-2.8660\times 10^{4}$
	Std.	$7.2056\times 10^{2}$	$4.8328\times 10^{2}$	$1.4425\times 10^{3}$	$4.9624\times 10^{3}$	$8.7650\times 10^{-1}$	$8.6947\times 10^{2}$	$3.0978\times 10^{3}$	$1.5604\times 10^{2}$	$6.4969\times 10^{2}$	$1.0110\times 10^{3}$
	Rank	2	1	5	10	7	9	4	3	6	8
$f_{9}$	Mean	$0.0000\times 10^{0}$	$1.1293\times 10^{2}$	$6.1389\times 10^{2}$	$7.4858\times 10^{2}$	$4.8001\times 10^{2}$	$7.2338\times 10^{2}$	$7.0295\times 10^{2}$	$5.4158\times 10^{2}$	$4.7545\times 10^{2}$	$1.6458\times 10^{3}$
	Std.	$0.0000\times 10^{0}$	$4.6876\times 10^{1}$	$6.6795\times 10^{1}$	$2.4301\times 10^{1}$	$2.3650\times 10^{-1}$	$1.0096\times 10^{2}$	$6.9653\times 10^{1}$	$4.1889\times 10^{1}$	$2.8058\times 10^{1}$	$3.7155\times 10^{1}$
	Rank	1	2	6	9	4	8	7	5	3	10
$f_{10}$	Mean	$4.4408\times 10^{-16}$	$4.4409\times 10^{-16}$	$2.3058\times 10^{0}$	$1.5183\times 10^{1}$	$1.7293\times 10^{1}$	$1.8159\times 10^{1}$	$1.7544\times 10^{1}$	$1.7654\times 10^{1}$	$2.4297\times 10^{0}$	$2.0361\times 10^{1}$
	Std.	$1.0029\times 10^{-31}$	$1.0029\times 10^{-31}$	$2.5542\times 10^{-1}$	$5.7627\times 10^{-1}$	$9.7400\times 10^{-2}$	$6.7775\times 10^{-2}$	$1.6684\times 10^{-1}$	$2.9820\times 10^{0}$	$3.8545\times 10^{-2}$	$1.4256\times 10^{-1}$
	Rank	1	1	3	5	6	9	7	8	4	10
$f_{11}$	Mean	$0.0000\times 10^{0}$	$0.0000\times 10^{0}$	$7.4240\times 10^{-3}$	$3.2412\times 10^{3}$	$4.8015\times 10^{3}$	$4.9370\times 10^{3}$	$1.8074\times 10^{2}$	$1.1910\times 10^{-3}$	$1.7048\times 10^{0}$	$3.3068\times 10^{3}$
	Std.	$0.0000\times 10^{0}$	$0.0000\times 10^{0}$	$6.5100\times 10^{-3}$	$1.3749\times 10^{2}$	$8.5320\times 10^{-1}$	$2.6842\times 10^{2}$	$3.6084\times 10^{1}$	$1.1480\times 10^{-3}$	$1.4301\times 10^{-2}$	$1.1330\times 10^{2}$
	Rank	1	1	4	7	9	10	6	3	5	8
$f_{12}$	Mean	$1.1938\times 10^{-1}$	$1.4009\times 10^{-1}$	$5.3982\times 10^{0}$	$4.0700\times 10^{5}$	$1.0000\times 10^{8}$	$1.6900\times 10^{9}$	$4.3700\times 10^{7}$	$1.0000\times 10^{10}$	$2.3426\times 10^{1}$	$8.1400\times 10^{9}$
	Std.	$1.9587\times 10^{-2}$	$9.6674\times 10^{-2}$	$5.9591\times 10^{-1}$	$4.7700\times 10^{5}$	$1.9900\times 10^{-5}$	$4.2800\times 10^{8}$	$3.2200\times 10^{7}$	$4.5000\times 10^{-3}$	$5.5985\times 10^{-1}$	$9.5400\times 10^{8}$
	Rank	1	2	3	5	7	8	6	10	4	9
Average		1.1667	3.0000	3.8333	6.1667	7.0833	7.7500	6.5833	5.2500	5.2500	8.6667
Overall		1	2	3	6	8	9	7	4	4	10

**Table 11:** Statistical comparison between pIPA and other algorithms for 200-dimensional problems.

pIPA vs.	*Z*-val.	R+	R−	ρ-val.	Sign.
IPA	−2.1915	6	22	0.0285	pIPA
ALO	−2.4318	8	70	0.0151	pIPA
PSO	−3.0594	0	78	0.0022	pIPA
SMS	−3.0594	0	78	0.0022	pIPA
BA	−3.0594	0	78	0.0022	pIPA
FPA	−3.0594	0	78	0.0022	pIPA
CS	−3.0594	0	78	0.0022	pIPA
FA	−3.0594	0	78	0.0022	pIPA
GA	−3.0594	0	78	0.0022	pIPA

**Table 12:** CEC 2015 benchmark functions used in experiments.

Fn.	Name	Related Basic Functions	Min.
$f_{1}$	Rotated Bent Cigar Function	Bent Cigar function	100
$f_{2}$	Rotated Discus Function	Discus function	200
$f_{3}$	Shifted and Rotated Weierstrass function	Weierstrass function	300
$f_{4}$	Shifted and Rotated Katsuura function	Katsuura function	500
$f_{5}$	Shifted and Rotated HappyCat function	HappyCat function	600
$f_{6}$	Shifted and Rotated HGBat function	HGBat function	700
$f_{7}$	Shifted and Rotated Expanded Griewank’s plus Rosenbrock’s function	Griewank’s function Rosenbrock’s function	800
$f_{8}$	Shifted and Rotated Expanded Scaffer’s $f_{6}$ function	Expanded Scaffer’s $f_{6}$ function	900
$f_{9}$	Hybrid function 2 (N = 4)	Griewank’s function Weierstrass function Rosenbrock’s function Scaffer’s $f_{6}$ function	1100
$f_{10}$	Compositional function 2 (N = 3)	Schwefel’s function Rastrigin’s function High conditioned elliptic	1400

**Table 13:** Results of pIPA with different prc values for CEC 2015 problems.

Fn.		prc								
		**30**	**35**	**40**	**50**	**60**	**70**	**80**	**90**	**95**
$f_{1}$	Mean	$3.7533\times 10^{10}$	$2.9575\times 10^{10}$	$2.0975\times 10^{10}$	$1.3501\times 10^{8}$	$8.2414\times 10^{7}$	$5.9617\times 10^{7}$	$4.5641\times 10^{6}$	$3.4240\times 10^{6}$	$4.1827\times 10^{7}$
	Std.	$2.3379\times 10^{9}$	$1.6950\times 10^{9}$	$1.8107\times 10^{9}$	$3.6707\times 10^{7}$	$2.8997\times 10^{7}$	$2.3105\times 10^{7}$	$1.4370\times 10^{5}$	$1.1789\times 10^{5}$	$1.8977\times 10^{7}$
$f_{2}$	Mean	$5.5678\times 10^{4}$	$5.5316\times 10^{4}$	$5.5047\times 10^{4}$	$4.8000\times 10^{4}$	$4.6881\times 10^{4}$	$4.1987\times 10^{4}$	$3.8121\times 10^{4}$	$3.1553\times 10^{4}$	$2.7425\times 10^{4}$
	Std.	$2.2628\times 10^{3}$	$2.6524\times 10^{3}$	$3.7518\times 10^{3}$	$6.0388\times 10^{3}$	$5.1357\times 10^{3}$	$4.8650\times 10^{3}$	$3.9484\times 10^{3}$	$4.0584\times 10^{3}$	$3.8092\times 10^{3}$
$f_{3}$	Mean	$3.3521\times 10^{2}$	$3.3408\times 10^{2}$	$3.3278\times 10^{2}$	$3.2453\times 10^{2}$	$3.2029\times 10^{2}$	$3.2089\times 10^{2}$	$3.2511\times 10^{2}$	$3.2575\times 10^{2}$	$3.2651\times 10^{2}$
	Std.	$6.7262\times 10^{-1}$	$6.2092\times 10^{-1}$	$9.7994\times 10^{-1}$	$1.6189\times 10^{0}$	$1.8962\times 10^{0}$	$2.2909\times 10^{0}$	$1.9613\times 10^{0}$	$2.1401\times 10^{0}$	$2.5209\times 10^{0}$
$f_{4}$	Mean	$5.0261\times 10^{2}$	$5.0268\times 10^{2}$	$5.0276\times 10^{2}$	$5.0156\times 10^{2}$	$5.0157\times 10^{2}$	$5.0145\times 10^{2}$	$5.0153\times 10^{2}$	$5.0147\times 10^{2}$	$5.0161\times 10^{2}$
	Std.	$3.5864\times 10^{-1}$	$3.3103\times 10^{-1}$	$3.6355\times 10^{-1}$	$2.0925\times 10^{-1}$	$2.1087\times 10^{-1}$	$2.4310\times 10^{-1}$	$2.2454\times 10^{-1}$	$2.7693\times 10^{-1}$	$2.5106\times 10^{-1}$
$f_{5}$	Mean	$6.0469\times 10^{2}$	$6.0415\times 10^{2}$	$6.0360\times 10^{2}$	$6.0035\times 10^{2}$	$6.0038\times 10^{2}$	$6.0040\times 10^{2}$	$6.0042\times 10^{2}$	$6.0047\times 10^{2}$	$6.0049\times 10^{2}$
	Std.	$1.4170\times 10^{-1}$	$1.6344\times 10^{-1}$	$1.4724\times 10^{-1}$	$4.7426\times 10^{-2}$	$4.0651\times 10^{-2}$	$5.9554\times 10^{-2}$	$5.4631\times 10^{-2}$	$6.6338\times 10^{-2}$	$8.6378\times 10^{-2}$
$f_{6}$	Mean	$7.7346\times 10^{2}$	$7.5805\times 10^{2}$	$7.4284\times 10^{2}$	$7.0029\times 10^{2}$	$7.0029\times 10^{2}$	$7.0030\times 10^{2}$	$7.0030\times 10^{2}$	$7.0033\times 10^{2}$	$7.0035\times 10^{2}$
	Std.	$4.3267\times 10^{0}$	$3.5335\times 10^{0}$	$3.8707\times 10^{0}$	$3.2153\times 10^{-2}$	$3.4482\times 10^{-2}$	$3.0737\times 10^{-2}$	$2.9642\times 10^{-2}$	$4.1421\times 10^{-2}$	$1.1513\times 10^{-1}$
$f_{7}$	Mean	$9.8282\times 10^{5}$	$5.9951\times 10^{5}$	$2.2924\times 10^{5}$	$8.5259\times 10^{2}$	$8.4316\times 10^{2}$	$8.4540\times 10^{2}$	$8.3899\times 10^{2}$	$8.4464\times 10^{2}$	$8.4764^{2}$
	Std.	$1.9916\times 10^{5}$	$1.9050\times 10^{5}$	$1.0964\times 10^{5}$	$1.1751\times 10^{1}$	$8.4363\times 10^{0}$	$6.5648\times 10^{0}$	$7.3209\times 10^{0}$	$1.1603\times 10^{1}$	$1.5102\times 10^{1}$
$f_{8}$	Mean	$9.1330\times 10^{2}$	$9.1326\times 10^{2}$	$9.1314\times 10^{2}$	$9.1252\times 10^{2}$	$9.1256\times 10^{2}$	$9.1264\times 10^{2}$	$9.1246\times 10^{2}$	$9.1237\times 10^{2}$	$9.1251\times 10^{2}$
	Std.	$1.7393\times 10^{-1}$	$1.4398\times 10^{-1}$	$2.3888\times 10^{-1}$	$4.1683\times 10^{-1}$	$2.7116\times 10^{-1}$	$2.7380\times 10^{-1}$	$4.4246\times 10^{-1}$	$4.0923\times 10^{-1}$	$4.9238\times 10^{-1}$
$f_{9}$	Mean	$1.2639\times 10^{3}$	$1.2533\times 10^{3}$	$1.2340\times 10^{3}$	$1.1228\times 10^{3}$	$1.1222\times 10^{3}$	$1.1222\times 10^{3}$	$1.1225\times 10^{3}$	$1.1223\times 10^{3}$	$1.1228\times 10^{3}$
	Std.	$1.0271\times 10^{1}$	$5.6144\times 10^{0}$	$1.3142\times 10^{1}$	$1.2191\times 10^{0}$	$1.2990\times 10^{0}$	$1.5804\times 10^{0}$	$1.4814\times 10^{0}$	$1.7848\times 10^{0}$	$2.1571\times 10^{0}$
$f_{10}$	Mean	$1.7523\times 10^{3}$	$1.7371\times 10^{3}$	$1.7177\times 10^{3}$	1.6323e+03	$1.6319\times 10^{3}$	$1.6327\times 10^{3}$	$1.6318\times 10^{3}$	$1.6319\times 10^{3}$	$1.6227\times 10^{3}$
	Std.	$1.2547\times 10^{1}$	$9.8808\times 10^{0}$	$1.1279\times 10^{1}$	$6.5722\times 10^{0}$	$5.8315\times 10^{0}$	$4.2383\times 10^{0}$	$5.6273\times 10^{0}$	$5.8416\times 10^{0}$	$4.4104\times 10^{0}$

**Table 14:** Comparison between pIPA and other meta-heuristics for CEC 2015 problems.

Fn.		pIPA	IPA	SOA	SHO	GWO	PSO	MFO	MVO	SCA	GSA	GA	DE
$f_{1}$	Mean	$3.42\times 10^{6}$	$1.28\times 10^{6}$	$2.55\times 10^{5}$	$2.28\times 10^{6}$	$2.02\times 10^{6}$	$4.37\times 10^{5}$	$1.47\times 10^{6}$	$6.06\times 10^{5}$	$7.65\times 10^{6}$	$3.20\times 10^{7}$	$8.89\times 10^{6}$	$6.09\times 10^{6}$
	Std.	$1.17\times 10^{5}$	$4.46\times 10^{5}$	$2.45\times 10^{5}$	$2.18\times 10^{6}$	$2.01\times 10^{6}$	$1.81\times 10^{5}$	$1.00\times 10^{6}$	$5.02\times 10^{5}$	$3.07\times 10^{6}$	$2.98\times 10^{6}$	$6.95\times 10^{6}$	$5.11\times 10^{6}$
	Rank	8	4	1	7	6	2	5	3	10	12	11	9
$f_{2}$	Mean	$2.74\times 10^{4}$	$5.46\times 10^{4}$	$5.53\times 10^{6}$	$3.13\times 10^{5}$	$5.65\times 10^{6}$	$9.41\times 10^{3}$	$1.97\times 10^{4}$	$1.43\times 10^{4}$	$7.33\times 10^{8}$	$4.58\times 10^{3}$	$2.97\times 10^{5}$	$4.40\times 10^{4}$
	Std.	$3.80\times 10^{3}$	$1.01\times 10^{4}$	$8.37\times 10^{4}$	$2.10\times 10^{5}$	$2.19\times 10^{6}$	$4.82\times 10^{3}$	$1.46\times 10^{4}$	$1.03\times 10^{4}$	$2.33\times 10^{8}$	$1.09\times 10^{3}$	$2.85\times 10^{3}$	$2.75\times 10^{4}$
	Rank	5	7	10	9	11	2	4	3	12	1	8	6
$f_{3}$	Mean	$3.20\times 10^{2}$	$3.20\times 10^{2}$	$3.20\times 10^{2}$	$3.20\times 10^{2}$	$3.20\times 10^{2}$	$3.20\times 10^{2}$	$3.20\times 10^{2}$	$3.20\times 10^{2}$	$3.20\times 10^{2}$	$3.20\times 10^{2}$	$3.20\times 10^{2}$	$3.20\times 10^{2}$
	Std.	$1.89\times 10^{0}$	$7.21\times 10^{-3}$	$3.71\times 10^{-3}$	$3.76\times 10^{-2}$	$7.08\times 10^{-2}$	$8.61\times 10^{-2}$	$9.14\times 10^{-2}$	$3.19\times 10^{-2}$	$7.53\times 10^{-2}$	$1.02\times 10^{-3}$	$2.78\times 10^{-2}$	$1.15\times 10^{-3}$
	Rank	1	1	1	1	1	1	1	1	1	1	1	1
$f_{4}$	Mean	$5.01\times 10^{2}$	$5.01\times 10^{2}$	$9.54\times 10^{2}$	$9.13\times 10^{2}$	$9.20\times 10^{2}$	$8.65\times 10^{2}$	$1.33\times 10^{3}$	$1.09\times 10^{3}$	1.76 × 10^3^	$1.75\times 10^{3}$	$1.26\times 10^{3}$	$3.34\times 10^{3}$
	Std.	$2.43\times 10^{-1}$	$1.64\times 10^{-1}$	$2.12\times 10^{2}$	$1.85\times 10^{2}$	$1.78\times 10^{2}$	$2.16\times 10^{2}$	$3.45\times 10^{2}$	$2.81\times 10^{2}$	$2.30\times 10^{2}$	$2.79\times 10^{2}$	$1.86\times 10^{2}$	$3.01\times 10^{2}$
	Rank	1	1	6	4	5	3	9	7	11	10	8	12
$f_{5}$	Mean	$6.00\times 10^{2}$	$6.00\times 10^{2}$	$2.47\times 10^{3}$	$1.29\times 10^{4}$	$2.26\times 10^{4}$	$1.86\times 10^{3}$	$7.35\times 10^{3}$	$3.82\times 10^{3}$	2.30 × 10^4^	$3.91\times 10^{6}$	$2.91\times 10^{5}$	$5.39\times 10^{4}$
	Std.	$4.74\times 10^{-2}$	$1.91\times 10^{-2}$	$1.40\times 10^{3}$	$1.15\times 10^{4}$	$2.07\times 10^{4}$	$1.28\times 10^{3}$	$3.82\times 10^{3}$	$2.44\times 10^{3}$	$1.91\times 10^{4}$	$2.70\times 10^{6}$	$1.67\times 10^{5}$	$2.40\times 10^{4}$
	Rank	1	1	4	7	9	3	6	5	8	12	11	10
$f_{6}$	Mean	$7.00\times 10^{2}$	$7.00\times 10^{2}$	$7.02\times 10^{2}$	$7.02\times 10^{2}$	$7.02\times 10^{2}$	$7.02\times 10^{2}$	$7.02\times 10^{2}$	$7.02\times 10^{2}$	$7.06\times 10^{2}$	$7.08\times 10^{2}$	$7.08\times 10^{2}$	$7.06\times 10^{2}$
	Std.	$3.21\times 10^{-2}$	$2.71\times 10^{-2}$	$5.76\times 10^{-1}$	$6.76\times 10^{-1}$	$7.07\times 10^{-1}$	$7.75\times 10^{-1}$	$1.10\times 10^{0}$	$9.40\times 10^{-1}$	$9.07\times 10^{-1}$	$1.32\times 10^{0}$	$2.97\times 10^{0}$	$3.87\times 10^{0}$
	Rank	1	1	3	3	3	3	3	3	9	12	12	9
$f_{7}$	Mean	$8.38\times 10^{2}$	$8.27\times 10^{2}$	$1.65\times 10^{3}$	$1.86\times 10^{3}$	$3.49\times 10^{3}$	$3.43\times 10^{3}$	$9.93\times 10^{3}$	$2.58\times 10^{3}$	$6.73\times 10^{3}$	$6.07\times 10^{5}$	$5.79\times 10^{4}$	$5.60\times 10^{3}$
	Std.	$7.32\times 10^{0}$	$3.86\times 10^{0}$	$2.12\times 10^{3}$	$1.98\times 10^{3}$	$2.04\times 10^{3}$	$2.77\times 10^{3}$	$8.74\times 10^{3}$	$1.61\times 10^{3}$	$3.36\times 10^{3}$	$4.81\times 10^{5}$	$2.76\times 10^{4}$	$5.15\times 10^{4}$
	Rank	2	1	3	4	7	6	10	5	9	12	11	8
$f_{8}$	Mean	$9.12\times 10^{2}$	$9.12\times 10^{2}$	$1.00\times 10^{3}$	$1.00\times 10^{3}$	$1.00\times 10^{3}$	$1.00\times 10^{3}$	$1.00\times 10^{3}$	$1.00\times 10^{3}$	$1.00\times 10^{3}$	$1.00\times 10^{3}$	$1.00\times 10^{3}$	$1.00\times 10^{3}$
	Std.	$4.09\times 10^{-1}$	$2.10\times 10^{-1}$	$7.35\times 10^{-1}$	$1.43\times 10^{-1}$	$1.28\times 10^{-1}$	$7.23\times 10^{-2}$	$2.20\times 10^{-1}$	$5.29\times 10^{-2}$	$9.79\times 10^{-1}$	$5.33\times 10^{0}$	$3.97\times 10^{0}$	$4.05\times 10^{0}$
	Rank	1	1	3	3	3	3	3	3	3	3	3	3
$f_{9}$	Mean	$1.12\times 10^{3}$	$1.12\times 10^{3}$	$1.32\times 10^{3}$	$1.38\times 10^{3}$	$1.40\times 10^{3}$	$1.35\times 10^{3}$	$1.37\times 10^{3}$	$1.39\times 10^{3}$	$1.35\times 10^{3}$	$1.41\times 10^{3}$	$1.36\times 10^{3}$	$1.65\times 10^{3}$
	Std.	$1.29\times 10^{0}$	$1.29\times 10^{0}$	$1.34\times 10^{1}$	$2.42\times 10^{1}$	$5.81\times 10^{1}$	$1.12\times 10^{2}$	$8.97\times 10^{1}$	$5.42\times 10^{1}$	$1.11\times 10^{2}$	$7.73\times 10^{1}$	$5.39\times 10^{1}$	$3.00\times 10^{1}$
	Rank	1	1	3	8	10	4	7	9	4	11	6	12
$f_{10}$	Mean	$1.62\times 10^{3}$	$1.62\times 10^{3}$	$3.78\times 10^{3}$	$4.25\times 10^{3}$	$7.29\times 10^{3}$	$7.10\times 10^{3}$	$7.60\times 10^{3}$	$7.34\times 10^{3}$	$7.51\times 10^{3}$	$9.30\times 10^{3}$	$8.96\times 10^{3}$	$6.08\times 10^{3}$
	Std.	$4.41\times 10^{0}$	$4.09\times 10^{0}$	$2.32\times 10^{3}$	$1.73\times 10^{3}$	$2.45\times 10^{3}$	$3.12\times 10^{3}$	$1.29\times 10^{3}$	$2.47\times 10^{3}$	$1.52\times 10^{3}$	$1.94\times 10^{3}$	$6.32\times 10^{3}$	$5.83\times 10^{3}$
	Rank	1	1	3	4	7	6	10	8	9	12	11	5
Average		2.200	1.900	3.700	5.000	6.200	3.300	5.800	4.700	7.600	8.600	8.200	7.500
Overall		2	1	4	6	8	3	7	5	10	12	11	9

**Table 15:** Results of pIPA with different prc values for D4, D4N, D12 and D12N instances.

Ins.		prc								
		**30**	**35**	**40**	**50**	**60**	**70**	**80**	**90**	**95**
D4	Mean	$1.8513\times 10^{0}$	$1.7988\times 10^{0}$	$1.7444\times 10^{0}$	$1.6592\times 10^{0}$	$1.6571\times 10^{0}$	$1.6330\times 10^{0}$	$1.6211\times 10^{0}$	$1.6393\times 10^{0}$	$1.6320\times 10^{0}$
	Std.	$6.5158\times 10^{-2}$	$4.6477\times 10^{-2}$	$5.1960\times 10^{-2}$	$6.2309\times 10^{-2}$	$5.0344\times 10^{-2}$	$6.0292\times 10^{-2}$	$5.5865\times 10^{-2}$	$4.9579\times 10^{-2}$	$5.6859\times 10^{-2}$
	Best	$1.6644\times 10^{0}$	$1.6852\times 10^{0}$	$1.6513\times 10^{0}$	$1.5322\times 10^{0}$	$1.5596\times 10^{0}$	$1.4706\times 10^{0}$	$1.5267\times 10^{0}$	$1.5120\times 10^{0}$	$1.5268\times 10^{0}$
D4N	Mean	$1.8411\times 10^{0}$	$1.7952\times 10^{0}$	$1.7555\times 10^{0}$	$1.6544\times 10^{0}$	$1.6514\times 10^{0}$	$1.6400\times 10^{0}$	$1.6070\times 10^{0}$	$1.6479\times 10^{0}$	$1.6454\times 10^{0}$
	Std.	$6.1375\times 10^{-2}$	$6.0277\times 10^{-2}$	$4.9922\times 10^{-2}$	$4.0500\times 10^{-2}$	$5.5370\times 10^{-2}$	$6.4636\times 10^{-2}$	$6.8443\times 10^{-2}$	$6.2579\times 10^{-2}$	$6.4412\times 10^{-2}$
	Best	$1.7299\times 10^{0}$	$1.6860\times 10^{0}$	$1.6111\times 10^{0}$	$1.5654\times 10^{0}$	$1.5498\times 10^{0}$	$1.5552\times 10^{0}$	$1.5015\times 10^{0}$	$1.5143\times 10^{0}$	$1.5018\times 10^{0}$
D12	Mean	$2.0227\times 10^{0}$	$1.9516\times 10^{0}$	$1.8930\times 10^{0}$	$1.8337\times 10^{0}$	$1.8138\times 10^{0}$	$1.8030\times 10^{0}$	$1.7883\times 10^{0}$	$1.7936\times 10^{0}$	$1.8140\times 10^{0}$
	Std.	$4.8096\times 10^{-2}$	$4.8999\times 10^{-2}$	$3.7801\times 10^{-2}$	$4.9484\times 10^{-2}$	$4.5847\times 10^{-2}$	$3.7111\times 10^{-2}$	$4.1546\times 10^{-2}$	$3.9991\times 10^{-2}$	$3.8952\times 10^{-2}$
	Best	$1.9097\times 10^{0}$	$1.8251\times 10^{0}$	$1.7666\times 10^{0}$	$1.7181\times 10^{0}$	$1.6842\times 10^{0}$	$1.6904\times 10^{0}$	$1.6904\times 10^{0}$	$1.6948\times 10^{0}$	$1.7433\times 10^{0}$
D12N	Mean	$2.0010\times 10^{0}$	$1.9527\times 10^{0}$	$1.8936\times 10^{0}$	$1.8423\times 10^{0}$	$1.8265\times 10^{0}$	1.8019 × 10^0^	1.7945 × 10^0^	$1.7913\times 10^{0}$	$1.8098\times 10^{0}$
	Std.	$5.1782\times 10^{-2}$	$4.0127\times 10^{-2}$	$2.8903\times 10^{-2}$	$3.9681\times 10^{-2}$	$3.9095\times 10^{-2}$	$4.0286\times 10^{-2}$	$3.9192\times 10^{-2}$	$4.5911\times 10^{-2}$	$4.9201\times 10^{-2}$
	Best	$1.9183\times 10^{0}$	$1.8721\times 10^{0}$	$1.8360\times 10^{0}$	$1.7365\times 10^{0}$	$1.7272\times 10^{0}$	$1.6730\times 10^{0}$	$1.7064\times 10^{0}$	$1.6781\times 10^{0}$	$1.7083\times 10^{0}$

**Table 16:** Comparison between pIPA and other algorithms for D4, D4N, D12 and D12N instances.

Ins.		pIPA	IPA	GA	PSO	DE	ABC	GSA	MFO	SCA	SSA	HHO
D4	Mean	$1.6211\times 10^{0}$	$1.6599\times 10^{0}$	$2.1300\times 10^{0}$	$7.8788\times 10^{0}$	$1.4654\times 10^{1}$	$2.0042\times 10^{1}$	$1.7590e\times 10^{0}$	$2.0462\times 10^{1}$	$6.0747\times 10^{0}$	$2.7771\times 10^{0}$	$1.7433\times 10^{0}$
	Best	$1.5267\times 10^{0}$	$1.5458\times 10^{0}$	$1.8930\times 10^{0}$	$7.2433\times 10^{0}$	$7.4238\times 10^{0}$	$1.8982\times 10^{1}$	$1.6384\times 10^{0}$	$1.9546\times 10^{1}$	$3.0410\times 10^{0}$	$2.6027\times 10^{0}$	$1.5979\times 10^{0}$
	Std.	$5.5865\times 10^{-2}$	$6.1728\times 10^{-2}$	$1.5558\times 10^{-1}$	$2.7104\times 10^{-1}$	$6.3289\times 10^{0}$	$4.2809\times 10^{-1}$	$7.9340\times 10^{-2}$	$3.1975\times 10^{-1}$	$1.4271\times 10^{0}$	$1.1751\times 10^{-1}$	$5.0111\times 10^{-2}$
	Rank	1	2	5	8	9	10	4	11	7	6	3
D4N	Mean	$1.6070\times 10^{0}$	$1.6989\times 10^{0}$	$2.1491\times 10^{0}$	$7.8962\times 10^{0}$	$1.6886\times 10^{1}$	$2.0101\times 10^{1}$	$1.7588\times 10^{0}$	$2.0361\times 10^{1}$	$6.4380\times 10^{0}$	$2.8256\times 10^{0}$	$1.75576\times 10^{0}$
	Best	$1.5015\times 10^{0}$	$1.5871\times 10^{0}$	$1.8715\times 10^{0}$	$7.5527\times 10^{0}$	$5.3421\times 10^{0}$	$1.9608\times 10^{1}$	$1.5660\times 10^{0}$	$1.9852\times 10^{1}$	$3.5316\times 10^{0}$	$2.6706\times 10^{0}$	$1.6424\times 10^{0}$
	Std.	$6.8443\times 10^{-2}$	$5.2602\times 10^{-2}$	$1.5647\times 10^{-1}$	$2.2522\times 10^{-1}$	$7.0483\times 10^{0}$	$3.2692\times 10^{-1}$	$8.6096\times 10^{-2}$	$3.5109\times 10^{-1}$	$1.0361\times 10^{0}$	$8.5527\times 10^{-2}$	$4.2976\times 10^{-2}$
	Rank	1	2	5	8	9	10	4	11	7	6	3
D12	Mean	$1.7883\times 10^{0}$	$1.8370\times 10^{0}$	$2.8016\times 10^{0}$	$1.0634\times 10^{1}$	$2.2396\times 10^{1}$	$2.1958\times 10^{1}$	$2.1962\times 10^{0}$	$2.2027\times 10^{1}$	$6.9154\times 10^{0}$	$3.0998\times 10^{0}$	$1.8565\times 10^{0}$
	Best	$1.6904\times 10^{0}$	$1.7710\times 10^{0}$	$2.5454\times 10^{0}$	$1.0024\times 10^{1}$	$2.1890\times 10^{1}$	$2.1599\times 10^{1}$	$2.0587\times 10^{0}$	$2.1819\times 10^{1}$	$5.8082\times 10^{0}$	$2.9668\times 10^{0}$	$1.7988\times 10^{0}$
	Std.	$4.1546\times 10^{-2}$	$3.8295\times 10^{-2}$	$1.5078\times 10^{-1}$	$1.9250\times 10^{-1}$	$2.0155\times 10^{-1}$	$1.9688\times 10^{-1}$	$5.6204\times 10^{-2}$	$1.2472\times 10^{-1}$	$4.6994\times 10^{-1}$	$7.3073\times 10^{-2}$	$3.1295\times 10^{-2}$
	Rank	1	2	5	8	11	9	4	10	7	6	3
D12N	Mean	$1.7913\times 10^{0}$	$1.8359\times 10^{0}$	$2.7769\times 10^{0}$	$1.0613\times 10^{1}$	$2.2439\times 10^{1}$	$2.1954\times 10^{1}$	$2.2106\times 10^{0}$	$2.1970\times 10^{1}$	$6.8059\times 10^{0}$	$3.1211\times 10^{0}$	$1.8606\times 10^{0}$
	Best	$1.6781\times 10^{0}$	$1.7076\times 10^{0}$	$2.3848\times 10^{0}$	$1.0271\times 10^{1}$	$2.2020\times 10^{1}$	$2.1431\times 10^{1}$	$2.1154\times 10^{0}$	$2.1394\times 10^{1}$	$2.3134\times 10^{0}$	$2.9541\times 10^{0}$	$1.8106\times 10^{0}$
	Std.	$4.5911\times 10^{-2}$	$5.2990\times 10^{-2}$	$1.5087\times 10^{-1}$	$1.5696\times 10^{-1}$	$1.7023\times 10^{-1}$	$2.2401\times 10^{-1}$	$4.3377\times 10^{-2}$	$2.2827\times 10^{-1}$	$1.0449\times 10^{0}$	$7.7361\times 10^{-2}$	$3.3679\times 10^{-2}$
	Rank	1	2	5	8	11	9	4	10	7	6	3
Average		1	2	5	8	10	9.5	4	10.5	7	6	3
Overall		1	2	5	8	10	9	4	11	7	6	3

**Table 17:** Average execution times of pIPA, IPA and ABC for D4, D4N, D12 and D12N instances.

Ins.		pIPA			IPA	ABC
		prc				
		**50**	**80**	**90**		
D4	Best	4.084	3.834	3.710	3.454	3.886
	Worst	4.213	3.931	3.829	3.579	6.985
	Mean	4.168	3.897	3.775	3.479	4.469
	Std.	0.030	0.025	0.030	0.030	0.999
D4N	Best	4.160	3.924	3.742	3.456	3.745
	Worst	4.368	4.480	4.139	3.563	6.283
	Mean	4.275	4.132	3.904	3.478	4.771
	Std.	0.038	0.130	0.084	0.019	0.849
D12	Best	29.207	28.529	28.506	27.605	29.245
	Worst	29.207	39.307	29.089	33.195	39.852
	Mean	29.336	30.017	28.698	28.088	32.133
	Std.	0.121	2.172	0.113	1.303	2.417
D12N	Best	31.527	28.583	28.567	27.592	28.337
	Worst	33.102	34.402	33.017	39.336	36.514
	Mean	31.773	31.120	29.714	30.095	31.042
	Std.	0.308	1.131	1.267	2.720	2.564

**Table 18:** Statistical comparison between pIPA and other algorithms for D4, D4N, D12 and D12N instances.

**pIPA vs.**	**D4**			**D4N**		
	**Z-val.**	**ρ-val.**	**Sign.**	**Z-val.**	**ρ-val.**	**Sign.**
IPA	−2.6019	0.0093	pIPA	−4.1651	<0.0001	pIPA
GA	−4.7821	<0.0001	pIPA	−4.7821	<0.0001	pIPA
PSO	−4.7821	<0.0001	pIPA	−4.7821	<0.0001	pIPA
DE	−4.7821	<0.0001	pIPA	−4.7821	<0.0001	pIPA
ABC	−4.7821	<0.0001	pIPA	−4.7821	<0.0001	pIPA
GSA	−4.5353	<0.0001	pIPA	−4.4119	<0.0001	pIPA
MFO	−4.7821	<0.0001	pIPA	−4.7821	<0.0001	pIPA
SCA	−4.7821	<0.0001	pIPA	−4.7821	<0.0001	pIPA
SSA	−4.7821	<0.0001	pIPA	−4.7821	<0.0001	pIPA
HHO	−4.5970	<0.0001	pIPA	−4.7204	<0.0001	pIPA
**pIPA vs.**	**D12**			**D12N**		
	**Z-val.**	**ρ-val.**	**Sign.**	**Z-val.**	**ρ-val.**	**Sign.**
IPA	−4.3913	<0.0001	pIPA	−3.1778	0.0014	pIPA
GA	−4.7821	<0.0001	pIPA	−4.7821	<0.0001	pIPA
PSO	−4.7821	<0.0001	pIPA	−4.7821	<0.0001	pIPA
DE	−4.7821	<0.0001	pIPA	−4.7821	<0.0001	pIPA
ABC	−4.7821	<0.0001	pIPA	−4.7821	<0.0001	pIPA
GSA	−4.7821	<0.0001	pIPA	−4.7821	<0.0001	pIPA
MFO	−4.7821	<0.0001	pIPA	−4.7821	<0.0001	pIPA
SCA	−4.7821	<0.0001	pIPA	−4.7821	<0.0001	pIPA
SSA	−4.7821	<0.0001	pIPA	−4.7821	<0.0001	pIPA
HHO	−4.6176	<0.0001	pIPA	−4.5765	<0.0001	pIPA

**Table 19:** Information about the battlefield.

Threat	Location	Radius	Degree	Start	Target
1	(45,50)	10	2	(10,10)	(55,100)
2	(12,40)	10	10		
3	(32,68)	8	1		
4	(36,26)	12	2		
5	(55,80)	9	3		

**Table 20:** Results of pIPA with different prc values for path planning.

D		prc					
		**50**	**60**	**70**	**80**	**90**	**95**
5	Best	50.7012	50.3814	50.3811	50.3864	50.3861	50.3849
	Worst	51.3094	50.3940	50.3905	50.3899	50.3947	50.3897
	Mean	51.0057	50.3846	50.3843	50.3881	50.3908	50.3875
	Std.	0.0906	0.0027	0.0014	0.0015	0.0031	0.0017
10	Best	50.4067	50.3950	50.3830	50.3938	50.3793	50.3874
	Worst	50.4506	50.5045	50.4432	50.4686	50.4743	50.4544
	Mean	50.4297	50.4181	50.4098	50.4154	50.4310	50.4182
	Std.	0.0160	0.0332	0.0141	0.0221	0.0283	0.0216
15	Best	50.4046	50.4212	50.4547	50.4309	50.4161	50.4024
	Worst	50.9706	50.9718	51.0054	50.6137	62.9473	51.0377
	Mean	50.5132	50.5302	50.5909	50.5015	52.5018	50.6097
	Std.	0.1316	0.1646	0.1962	0.0497	4.4307	0.1953
20	Best	50.4996	50.5009	50.4637	50.4953	50.4386	50.5217
	Worst	51.0712	51.0393	68.8286	70.5922	70.9647	67.9162
	Mean	50.7875	50.7526	52.8225	53.7912	52.6790	52.4902
	Std.	0.2111	0.2029	5.6321	6.4586	5.6714	4.5896
25	Best	50.6205	50.5542	50.6268	50.5614	50.8930	50.6499
	Worst	51.0443	51.0518	51.2583	74.4010	82.6972	86.9844
	Mean	50.9344	50.9310	51.0171	52.0756	55.4928	56.0014
	Std.	0.0919	0.1584	0.1221	5.1497	9.5877	10.5172
30	Best	50.7012	50.6297	50.8999	50.9168	50.9879	50.9806
	Worst	51.3094	51.0698	78.2241	51.3553	81.7236	106.3330
	Mean	51.0057	50.9615	52.1351	51.0416	53.4306	61.0772
	Std.	0.0906	0.1188	5.3536	0.1038	8.1607	17.5799
35	Best	50.9957	50.9972	51.0092	50.9054	51.0118	51.0122
	Worst	51.3873	51.3815	51.0212	108.5633	119.0773	120.7746
	Mean	51.0391	51.0356	51.0139	55.4990	55.7945	62.5169
	Std.	0.0951	0.0820	0.0032	15.2441	15.5470	22.8805
40	Best	51.0032	50.9947	51.0068	51.0025	51.0053	50.9955
	Worst	51.2577	51.1638	51.4110	51.4374	132.1328	134.8970
	Mean	51.0572	51.0429	51.0844	51.0958	60.7355	76.0852
	Std.	0.0628	0.0420	0.0971	0.1274	24.9906	32.6241

**Table 21:** Comparison between pIPA and other meta-heuristics for path planning.

D		pIPA	IPA	ABC	BA	BAM	ACO	BBO	DE	ES	FA	GA	MFA	PBIL	PSO	SGA	PGSO
5	Best	50.385	50.384	50.384	60.690	54.357	61.372	60.330	54.357	59.590	54.359	55.247	54.357	59.763	55.167	55.654	53.380
	Worst	50.394	50.385	50.385	345.255	60.240	63.320	171.500	62.200	112.260	65.740	61.600	62.419	72.250	66.071	61.200	60.630
	Mean	50.384	50.384	50.384	106.483	59.054	61.520	72.730	58.596	80.720	58.750	60.470	59.167	66.139	59.906	60.501	53.669
	Std.	0.002	0.001	0.001	-	-	-	-	2.160	-	3.010	-	2.250	-	2.620	1.560	2.260
	Rank	1	1	1	16	7	12	14	5	15	6	10	8	13	9	11	4
10	Best	50.395	50.376	50.371	52.360	51.395	60.228	52.947	51.395	57.420	51.399	51.607	51.397	83.112	52.207	51.549	50.649
	Worst	50.504	50.457	50.406	108.738	60.7244	68.190	76.820	56.736	123.460	56.710	60.110	53.786	119.250	68.622	56.165	53.330
	Mean	50.418	50.398	50.384	69.425	52.707	61.950	57.965	53.104	76.280	52.180	52.542	51.574	101.440	57.041	52.279	50.849
	Std.	0.033	0.024	0.013	-	-	-	-	2.600	-	2.370	-	1.730	-	2.250	1.430	1.870
	Rank	3	2	1	14	9	13	12	10	15	6	8	5	16	11	7	4
15	Best	50.421	50.424	50.425	53.075	50.609	58.530	52.557	50.611	58.255	50.617	50.871	50.612	107.223	52.097	50.807	50.452
	Worst	50.971	51.219	50.789	85.745	60.192	61.000	90.370	62.580	103.860	94.276	57.447	53.832	189.200	87.320	61.800	55.460
	Mean	50.530	50.570	50.591	63.601	51.231	60.260	59.526	52.278	71.860	52.822	52.188	50.897	128.250	58.340	51.891	51.516
	Std.	0.164	0.193	0.100	-	-	-	-	3.730	-	4.250	-	1.340	-	4.010	2.450	1.490
	Rank	1	2	3	14	5	13	12	9	15	10	8	4	16	11	7	6
20	Best	50.500	50.495	50.866	52.395	50.467	60.445	54.723	50.510	60.232	50.463	50.825	50.455	130.152	52.464	50.846	50.657
	Worst	51.039	51.271	54.672	83.706	53.742	67.180	78.200	64.570	81.450	78.914	59.180	52.028	337.300	78.160	68.950	59.850
	Mean	50.752	50.925	52.181	63.630	50.760	66.220	61.88	52.722	70.190	53.733	53.090	50.700	185.430	58.248	53.167	52.398
	Std.	0.202	0.196	0.994	-	-	-	-	3.710	-	7.580	-	1.020	-	6.950	3.990	1.560
	Rank	2	4	5	13	3	14	12	7	15	10	8	1	16	11	9	6
25	Best	50.554	50.790	51.911	55.017	50.448	61.549	55.528	50.551	63.369	50.491	51.242	50.457	159.740	53.738	51.239	50.782
	Worst	51.051	57.314	57.530	74.926	53.519	62.070	80.330	69.660	83.910	66.452	60.398	53.704	699.600	78.139	65.700	63.160
	Mean	50.931	51.678	54.690	64.901	50.709	61.570	64.780	54.408	72.780	53.904	53.781	50.999	257.720	60.263	54.157	54.587
	Std.	0.158	1.503	1.618	-	-	-	-	4.120	-	8.660	-	0.810	-	7.550	4.060	2.380
	Rank	2	4	10	14	1	12	13	8	15	6	5	3	16	11	7	9
30	Best	50.629	50.997	54.527	57.247	50.467	63.230	56.607	50.898	65.725	50.683	51.921	50.516	230.150	53.299	51.617	51.019
	Worst	51.069	61.565	64.940	80.084	60.285	64.710	78.580	74.120	91.300	65.976	62.718	58.336	2396	93.695	64.710	75.320
	Mean	50.961	51.789	59.805	66.616	51.106	63.950	67.870	59.988	74.780	54.962	55.008	51.357	395.540	62.385	54.521	56.891
	Std.	0.118	2.275	2.366	-	-	-	-	6.740	-	9.120	-	1.230	-	8.200	4.110	3.450
	Rank	1	4	9	13	2	12	14	10	15	6	7	3	16	11	5	8
35	Best	50.997	51.005	57.259	57.448	50.479	66.960	63.021	52.537	66.745	51.083	52.311	50.471	270.330	55.503	51.633	54.136
	Worst	51.381	96.301	74.095	82.737	58.819	68.720	93.850	84.440	88.76	83.887	74.479	55.883	6362	82.833	67.610	71.450
	Mean	51.035	55.889	66.187	67.703	51.461	68.310	71.560	67.900	76.520	55.996	55.960	51.601	684.660	64.135	55.826	59.744
	Std.	0.082	12.249	4.298	-	-	-	-	9.150	-	9.550	-	1.650	-	8.650	4.120	4.010
	Rank	1	5	10	11	2	13	14	12	15	7	6	3	16	9	4	8
40	Best	50.994	51.025	63.269	58.650	50.602	69.795	63.550	54.549	68.231	51.523	52.208	50.561	390.620	55.737	52.618	55.092
	Worst	51.163	116.195	86.613	83.263	58.427	77.060	90.700	93.260	96.420	86.663	72.069	57.724	7103	84.730	67.870	72.650
	Mean	51.042	55.994	75.595	69.973	51.876	74.580	74.850	77.620	80.260	57.856	57.493	52.198	1169	64.885	57.110	62.420
	Std.	0.042	16.188	5.356	-	-	-	-	10.900	-	10.430	-	2.380	-	9.410	4.550	4.540
	Rank	1	4	13	10	2	11	12	14	15	7	6	3	16	9	5	8
Average		1.500	3.250	6.500	13.125	3.875	12.500	12.875	9.375	15.000	7.250	7.250	3.750	15.625	10.250	6.875	6.625
Overall		1	2	5	14	4	12	13	10	15	8	8	3	16	11	7	6

**Table 22:** Sr and Me metrics of pIPA, IPA, and ABC for path planning.

D		pIPA						IPA	ABC
		prc							
		**50**	**60**	**70**	**80**	**90**	**95**		
5	Sr	100.000	100.000	100.000	100.000	100.000	100.000	100.000	100.000
	Me	113.620	394.940	339.050	236.040	254.020	169.210	221.050	219.500
10	Sr	100.000	100.000	100.000	100.000	100.000	100.000	100.000	100.000
	Me	1690.840	1449.350	1143.660	1198.240	1661.700	1297.700	1605.580	825.500
15	Sr	100.000	100.000	100.000	100.000	84.000	100.000	100.000	100.000
	Me	2103.790	2147.470	1884.060	1944.260	2249.500	2387.500	2390.500	2120.000
20	Sr	100.000	100.000	88.000	81.000	90.000	88.000	100.000	100.000
	Me	2683.500	2409.040	2455.602	2483.790	2552.822	2891.705	3041.500	3854.500
25	Sr	100.000	100.000	100.000	95.000	81.000	80.000	92.000	59.000
	Me	2713.780	2749.150	2743.520	2600.653	2978.630	3248.750	4400.516	5126.000
30	Sr	100.000	100.000	96.000	100.000	92.000	74.000	90.000	5.000
	Me	2875.470	2716.710	2733.083	2932.460	3009.707	3083.135	4110.603	5910.000
35	Sr	100.000	100.000	100.000	92.000	91.000	78.000	83.000	0.000
	Me	3056.660	2968.200	3022.630	2743.663	3033.967	3478.551	4136.859	-
40	Sr	100.000	100.000	100.000	100.000	87.000	62.000	90.000	0.000
	Me	3213.640	3120.080	3054.880	3041.440	3533.655	3621.565	4526.558	-

**Table 23:** Statistical comparison between pIPA and other path planners.

**pIPA vs.**	**IPA**	**ABC**	**BA**	**BAM**	**ACO**
*Z*-val.	−1.9874	−2.4175	−2.6600	−0.9453	−2.6600
ρ-val.	0.0468	0.0156	0.0078	0.0685	0.0078
R+	2	1	0	17	0
R−	26	28	36	19	36
Sign.	pIPA	pIPA	pIPA	-	pIPA
**pIPA vs.**	**BBO**	**DE**	**ES**	**FA**	**GA**
*Z*-val.	−2.6600	−2.4175	−2.6600	−1.9213	−2.6600
ρ-val.	0.0078	0.0156	0.0078	0.0546	0.0078
R+	0	1	0	4	0
R−	36	35	36	32	36
Sign.	pIPA	pIPA	pIPA	-	pIPA
**pIPA vs.**	**MFA**	**PBIL**	**PSO**	**SGA**	**PGSO**
*Z*-val.	−0.0685	−2.6600	−2.6600	−2.6600	−2.6600
ρ-val.	0.9453	0.0078	0.0078	0.0078	0.0078
R+	17	0	0	0	0
R−	19	36	36	36	36
Sign.	-	pIPA	pIPA	pIPA	pIPA

## Data Availability

No new data were created or analyzed in this study.

## References

[1] Yong W., Tao W., Cheng-Zhi Z., Hua-Juan H. (2016). A new stochastic optimization approach—Dolphin swarm optimization algorithm. Int. J. Comput. Intell. Appl..

[2] Han C., Zhou G., Zhou Y. (2019). Binary symbiotic organism search algorithm for feature selection and analysis. IEEE Access.

[3] Xu M., Cao L., Lu D., Hu Z., Yue Y. (2023). Application of Swarm Intelligence Optimization Algorithms in Image Processing: A Comprehensive Review of Analysis, Synthesis, and Optimization. Biomimetics.

[4] Li J.Y., Zhan Z.H., Li Y., Zhang J. (2023). Multiple Tasks for Multiple Objectives: A New Multiobjective Optimization Method via Multitask Optimization. IEEE Trans. Evol. Comput..

[5] Peng H., Mei C., Zhang S., Luo Z., Zhang Q., Wu Z. (2023). Multi-strategy dynamic multi-objective evolutionary algorithm with hybrid environmental change responses. Swarm Evol. Comput..

[6] Srinivas M., Patnaik L.M. (1994). Genetic algorithms: A survey. Computer.

[7] Aslantas V., Toprak A.N. Multi focus image fusion by differential evolution algorithm. Proceedings of the 2014 11th International Conference on Informatics in Control, Automation and Robotics (ICINCO).

[8] Ahmad M.F., Isa N.A.M., Lim W.H., Ang K.M. (2022). Differential evolution: A recent review based on state-of-the-art works. Alex. Eng. J..

[9] Beyer H.G., Schwefel H.P. (2002). Evolution strategies–A comprehensive introduction. Nat. Comput..

[10] Baluja S. (1994). Population-Based Incremental Learning: A Method for Integrating Genetic Search Based Function Optimization and Competitive Learning.

[11] Simon D. (2008). Biogeography-based optimization. IEEE Trans. Evol. Comput..

[12] Zhang X., Wang D., Fu Z., Liu S., Mao W., Liu G., Jiang Y., Li S. (2020). Novel biogeography-based optimization algorithm with hybrid migration and global-best Gaussian mutation. Appl. Math. Model..

[13] Yue Y., Cao L., Lu D., Hu Z., Xu M., Wang S., Li B., Ding H. (2023). Review and empirical analysis of sparrow search algorithm. Artif. Intell. Rev..

[14] Dorigo M., Birattari M., Stutzle T. (2006). Ant colony optimization. IEEE Comput. Intell. Mag..

[15] Lu J., Zhang J., Sheng J. (2021). Enhanced multi-swarm cooperative particle swarm optimizer. Swarm Evol. Comput..

[16] Krishnanand K., Ghose D. (2009). Glowworm swarm optimisation: A new method for optimising multi-modal functions. Int. J. Comput. Intell. Stud..

[17] Yang X.S., Deb S. Cuckoo search via Levy flights. Proceedings of the 2009 World Congress on Nature & Biologically Inspired Computing (NaBIC).

[18] Yang X.S. (2010). Firefly algorithm, Levy flights and global optimization. Research and Development in Intelligent Systems XXVI.

[19] Yang X.S. (2010). A new metaheuristic bat-inspired algorithm. Nature Inspired Cooperative Strategies for Optimization (NICSO 2010).

[20] Yang X.S. (2012). Flower pollination algorithm for global optimization. Proceedings of the International Conference on Unconventional Computing and Natural Computation.

[21] Bansal J.C., Gopal A., Nagar A.K. (2018). Stability analysis of artificial bee colony optimization algorithm. Swarm Evol. Comput..

[22] Gul E., Toprak A.N. (2023). Contourlet and discrete cosine transform based quality guaranteed robust image watermarking method using artificial bee colony algorithm. Expert Syst. Appl..

[23] Mirjalili S., Mirjalili S.M., Lewis A. (2014). Grey wolf optimizer. Adv. Eng. Softw..

[24] Mirjalili S. (2015). Moth-flame optimization algorithm: A novel nature-inspired heuristic paradigm. Knowl.-Based Syst..

[25] Mirjalili S. (2015). The ant lion optimizer. Adv. Eng. Softw..

[26] Mirjalili S. (2016). SCA: A sine cosine algorithm for solving optimization problems. Knowl.-Based Syst..

[27] Mirjalili S., Mirjalili S.M., Hatamlou A. (2016). Multi-verse optimizer: A nature-inspired algorithm for global optimization. Neural Comput. Appl..

[28] Mirjalili S., Gandomi A.H., Mirjalili S.Z., Saremi S., Faris H., Mirjalili S.M. (2017). Salp Swarm Algorithm: A bio-inspired optimizer for engineering design problems. Adv. Eng. Softw..

[29] Heidari A.A., Mirjalili S., Faris H., Aljarah I., Mafarja M., Chen H. (2019). Harris hawks optimization: Algorithm and applications. Future Gener. Comput. Syst..

[30] Li S., Chen H., Wang M., Heidari A.A., Mirjalili S. (2020). Slime mould algorithm: A new method for stochastic optimization. Future Gener. Comput. Syst..

[31] Satapathy S., Naik A. (2016). Social group optimization (SGO): A new population evolutionary optimization technique. Complex Intell. Syst..

[32] Alimoradi M., Azgomi H., Asghari A. (2022). Trees Social Relations Optimization Algorithm: A new Swarm-Based metaheuristic technique to solve continuous and discrete optimization problems. Math. Comput. Simul..

[33] Pan J.S., Zhang L.G., Wang R.B., Snášel V., Chu S.C. (2022). Gannet optimization algorithm: A new metaheuristic algorithm for solving engineering optimization problems. Math. Comput. Simul..

[34] Kaveh M., Mesgari M.S., Saeidian B. (2023). Orchard Algorithm (OA): A new meta-heuristic algorithm for solving discrete and continuous optimization problems. Math. Comput. Simul..

[35] Dhiman G., Kumar V. (2017). Spotted hyena optimizer: A novel bio-inspired based metaheuristic technique for engineering applications. Adv. Eng. Softw..

[36] Dhiman G., Kumar V. (2018). Emperor penguin optimizer: A bio-inspired algorithm for engineering problems. Knowl.-Based Syst..

[37] Dhiman G., Kumar V. (2019). Seagull optimization algorithm: Theory and its applications for large-scale industrial engineering problems. Knowl.-Based Syst..

[38] Dhiman G., Kaur A. (2019). STOA: A bio-inspired based optimization algorithm for industrial engineering problems. Eng. Appl. Artif. Intell..

[39] Kaur S., Awasthi L.K., Sangal A., Dhiman G. (2020). Tunicate Swarm Algorithm: A new bio-inspired based metaheuristic paradigm for global optimization. Eng. Appl. Artif. Intell..

[40] Birbil I., Fang S.C. (2003). An electromagnetism-like mechanism for global optimization. J. Glob. Optim..

[41] Rashedi E., Nezamabadi-pour H., Saryazdi S. (2009). GSA: A Gravitational Search Algorithm. Inf. Sci..

[42] Formato R.A. (2009). Central force optimization: A new deterministic gradient-like optimization metaheuristic. Opsearch.

[43] Shen J., Li Y. Light ray optimization and its parameter analysis. Proceedings of the 2009 International Joint Conference on Computational Sciences and Optimization.

[44] Cuevas E., Echavarria A., Ramirez-Ortegon M.A. (2014). An optimization algorithm inspired by the States of Matter that improves the balance between exploration and exploitation. Appl. Intell..

[45] Javidy B., Hatamlou A., Mirjalili S. (2015). Ions motion algorithm for solving optimization problems. Appl. Soft Comput..

[46] Savsani P., Savsani V. (2016). Passing vehicle search (PVS): A novel metaheuristic algorithm. Appl. Math. Model..

[47] Azizi M. (2021). Atomic orbital search: A novel metaheuristic algorithm. Appl. Math. Model..

[48] Dehghani M., Trojovskỳ P., Malik O.P. (2023). Green Anaconda Optimization: A New Bio-Inspired Metaheuristic Algorithm for Solving Optimization Problems. Biomimetics.

[49] Trojovskỳ P., Dehghani M. (2023). Subtraction-Average-Based Optimizer: A New Swarm-Inspired Metaheuristic Algorithm for Solving Optimization Problems. Biomimetics.

[50] Trojovská E., Dehghani M., Leiva V. (2023). Drawer Algorithm: A New Metaheuristic Approach for Solving Optimization Problems in Engineering. Biomimetics.

[51] Aslan S., Demirci S. (2020). Immune Plasma Algorithm: A Novel Meta-Heuristic for Optimization Problems. IEEE Access.

[52] Langford E. (2006). Quartiles in Elementary Statistics. J. Stat. Educ..

[53] Schoonjans F., Bacquer D.D., Schmid P. (2011). Estimation of population percentiles. Epidemiology.

[54] Bornmann L., Leydesdorff L., Mutz R. (2013). The use of percentiles and percentile rank classes in the analysis of bibliometric data: Opportunities and limits. J. Inf..

[55] Eberhart R.C., Shi Y. Particle swarm optimization: Developments, applications and resources. Proceedings of the 2001 Congress on Evolutionary Computation.

[56] Price K.V. (2013). Differential evolution. Handbook of Optimization.

[57] Karaboga D., Basturk B. (2007). A powerful and efficient algorithm for numerical function optimization: Artificial bee colony (ABC) algorithm. J. Glob. Optim..

[58] Wang G.G., Guo L., Duan H., Liu L., Wang H. (2012). A bat algorithm with mutation for UCAV path planning. Sci. World J..

[59] Tang Z., Zhou Y. (2015). A glowworm swarm optimization algorithm for uninhabited combat air vehicle path planning. J. Intell. Syst..

[60] Wang G.G., Guo L., Duan H., Liu L., Wang H. (2012). A modified firefly algorithm for UCAV path planning. Int. J. Hybrid Inf. Technol..

[61] Chen Q., Liu B., Zhang Q., Liang J., Suganthan P., Qu B. Problem Definitions and Evaluation Criteria for CEC 2015 Special Session on Bound Constrained Single-Objective Computationally Expensive Numerical Optimization. Proceedings of the 2015 IEEE Congress on Evolutionary Computation (CEC).

[62] Fugini M., Finocchi J., Locatelli P. (2021). A Big Data Analytics Architecture for Smart Cities and Smart Companies. Big Data Res..

[63] Tang L., Li J., Du H., Li L., Wu J., Wang S. (2022). Big Data in Forecasting Research: A Literature Review. Big Data Res..

[64] Abbass H.A. Calibrating independent component analysis with Laplacian reference for real-time EEG artifact removal. Proceedings of the International Conference on Neural Information Processing.

[65] Goh S.K., Abbass H.A., Tan K.C., Al Mamun A. Artifact removal from EEG using a multi-objective independent component analysis model. Proceedings of the International Conference on Neural Information Processing.

[66] Goh S.K., Tan K.C., Al-Mamun A., Abbass H.A. Evolutionary big optimization (BigOpt) of signals. Proceedings of the 2015 IEEE Congress on Evolutionary Computation (CEC).

[67] Xu C., Duan H., Liu F. (2010). Chaotic artificial bee colony approach to Uninhabited Combat Air Vehicle (UCAV) path planning. Aerosp. Sci. Technol..

[68] Aslan S., Erkin T. (2023). An immune plasma algorithm based approach for UCAV path planning. J. King Saud-Univ.-Comput. Inf. Sci..

